# Enhancing reliability and security in cloud-based telesurgery systems leveraging swarm-evoked distributed federated learning framework to mitigate multiple attacks

**DOI:** 10.1038/s41598-025-12027-1

**Published:** 2025-07-26

**Authors:** S. Punitha, K. S. Preetha

**Affiliations:** School of Electronics Engineering, Vellore Institute of Technology, Vellore, India

**Keywords:** Telesurgery, Internet of things, Artificial intelligence, Optimized gated transformer networks, 5G/6G wireless communication, Internet of surgical things, Machine learning, Biomedical engineering, Health care

## Abstract

Advances in robotic surgery are being driven by the convergence of technologies such as artificial intelligence (AI), 5G/6G wireless communication, the Internet of Things (IoT), and edge computing, enhancing clinical precision, speed, and real-time decision-making. However, the practical deployment of telesurgery and tele-mentoring remains constrained due to increasing cybersecurity threats, posing significant challenges to patient safety and system reliability. To address these issues, a distributed framework based on federated learning is proposed, integrating Optimized Gated Transformer Networks (OGTN) with layered chaotic encryption schemes to mitigate multiple unknown cyberattacks while preserving data privacy and integrity. The framework was implemented using TensorFlow Federated Learning Libraries (FLL) and evaluated on the UNSW-NB15 dataset. Performance was assessed using metrics including precision, accuracy, F1-score, recall, and security strength, and compared with existing approaches. In addition, structured and unstructured security assessments, including evaluations based on National Institute of Standards and Technology (NIST) recommendations, were performed to validate robustness. The proposed framework demonstrated superior performance in terms of diagnostic accuracy and cybersecurity resilience relative to conventional models. These results suggest that the framework is a viable candidate for integration into teleoperated healthcare systems, offering improved security and operational efficiency in robotic surgery applications.

## Introduction

The field of robotic-assisted surgery is experiencing a ground-breaking evolution, fuelled by the infusion of advanced technologies, including artificial intelligence (AI), next-generation wireless networks (5G/6G), edge computing, and the Internet of Surgical Things (IoST)^1–3^. These innovations are redefining surgical procedures by enhancing accuracy, efficiency, and real-time clinical decision-making^4^. Telesurgery harmoniously incorporates robotic surgery with state-of-the-art communication technology, constituting remotely conducted surgical procedures with rapid data transmission across networks^5^. Within the realm of telesurgery, the robotic system maintains direct contact with the patient while the surgeon orchestrates the surgical procedure from a remote console. In this innovative approach, medical data, encompassing imaging, audio, and video modules, undergo digital conversion and are efficiently transmitted through wired or wireless communication channels^6–9^. Many medical robotic systems primarily rely on teleoperation as their main method of functionality. It is important to note that the master unit, also referred to as the expert site, and the slave robotic manipulator, known as the patient site, are often positioned within the same room^10–12^. Despite their close physical proximity, this form of surgery is classified as short distance telesurgery. In those scenarios, telerobotic systems are generally split into dual distinct locations: the local site, which includes the human operator along with all critical components required for remote system control (like display monitors, control interfaces like joysticks, keyboards, and varied input/output peripherals), and the remote site, which contains the robotic surgical system, the patient, and the supporting medical staff^13^. When implemented in surgical procedures, this technological approach is commonly referred to as telesurgery^14^.

Tele-surgery systems face distinct challenges, particularly the threat of illegal entry to confidential data and the manipulation of stored medical images^15^, . Given the highly sensitive nature of patient information, privacy concerns pose a significant complexity to the adoption of these systems. The deployment of intelligent surgical technologies may be constrained due to apprehensions regarding the sharing and accessibility of medical imagery. Consequently, ensuring robust security and safeguarding patient privacy are paramount^16–19^, . Recent research has explored the application of nature-inspired algorithms for optimizing service composition and resource allocation in cloud-based IoT environments. Table 1 provides a comparative overview of recent studies. For instance^20^, proposed a novel service composition method employing the Grey Wolf Optimization (GWO) algorithm in conjunction with the MapReduce framework to enhance computational efficiency and scalability in distributed IoT networks. Their work demonstrated how GWO’s hunting behaviour-based search strategy effectively reduced execution time and improved load balancing across services. The use of swarm intelligence in performance optimization has gained significant traction in the context of secure and distributed systems. Notably^21^, introduced a GSO-based multi‐objective optimization strategy tailored for blockchain-enabled Industrial Internet of Things (IIoT) applications. Their method effectively addressed conflicting objectives such as security, latency, and throughput by mimicking the luminescence-guided behaviour of glowworms. This bio-inspired algorithm demonstrated strong adaptability in heterogeneous IIoT environments, enhancing overall system responsiveness and robustness. The growing complexity of IoT environments, particularly in multi-user and latency-sensitive applications, necessitates advanced strategies for secure data offloading and model training. In this context^22^, proposed a robust solution that integrates blockchain technology with deep reinforcement learning (DRL) to manage secure and efficient offloading in IoT networks. Their framework ensures trust, data integrity, and low latency across multiple users by leveraging decentralized consensus mechanisms and adaptive learning. This work aligns closely with our federated learning-based approach, which aims to decentralize model training across multiple telesurgery nodes while safeguarding sensitive medical data. The main contribution of the paper by^23^ is its in-depth analysis of the reliability and availability of wireless sensor networks (WSNs) in industrial applications, with a specific focus on accounting for permanent faults. This work provides a robust framework for evaluating the performance of WSNs under real-world conditions, where sensor nodes might fail due to permanent faults, which are often neglected in conventional studies^24^. provide a comprehensive systematic literature review on the applications of nature-inspired algorithms in Internet of Things (IoT)-based healthcare services. It evaluates various algorithms, such as genetic algorithms, particle swarm optimization, and ant colony optimization, and their effectiveness in addressing challenges such as data management, optimization, and decision-making in IoT-based healthcare environments^25^. introduce a Gravitational Search Algorithm (GSO)-based multi-objective technique for optimizing the performance of blockchain-based IIoT systems. This work focuses on improving the key performance metrics such as throughput, latency, and security in blockchain-enabled IoT networks used in industrial settings. The proposed method leverages the GSO to efficiently solve optimization problems while balancing multiple objectives, which is crucial in environments where trade-offs between performance and security need to be carefully managed^26^. offer a comprehensive survey on the evolving threats posed by botnets and the defense strategies developed to mitigate these threats. It explores various botnet attack techniques, their impact on network systems, and the countermeasures available, including anomaly detection, machine learning-based defense mechanisms, and distributed denial-of-service (DDoS) attack prevention. The study emphasizes the importance of understanding these threats to develop robust security protocols, especially in the context of emerging technologies and complex network infrastructures^27^. present a novel nano-design for a quantum-based Arithmetic and Logic Unit (ALU) aimed at significantly enhancing the computational efficiency of future Internet of Things (IoT) applications. By integrating quantum computing principles into ALU architecture at the nanoscale, the study proposes a futuristic and energy-efficient approach to overcoming the limitations of classical computing in terms of speed, size, and power consumption. The design emphasizes scalability and suitability for advanced IoT systems that require high-performance processing under strict resource constraints^28^. introduces a nano-scale design of a Vedic multiplier tailored for electrocardiogram (ECG) signal processing, leveraging quantum technology to enhance computational speed and energy efficiency. The proposed design integrates ancient Vedic mathematical principles with modern quantum-based nano-architectures to achieve high-speed arithmetic operations essential for real-time biomedical signal analysis, particularly in resource-constrained and latency-sensitive applications like wearable or implantable medical devices^29^. focus on enhancing the analysis of solar convection phenomena using multi-core processors and graphics processing units (GPUs). The study demonstrates how parallel processing techniques significantly accelerate computational tasks and improve the accuracy and efficiency of complex simulations related to solar energy systems. By leveraging the high-performance capabilities of multi-core CPUs and GPUs, the authors present a scalable and efficient computational framework suitable for real-time scientific modeling. Similar performance gains can be achieved in telesurgical environments through the deployment of parallel computing strategies, thus reducing latency and improving the responsiveness and accuracy of surgical procedures conducted over networks^28^. presents a nano-scale Vedic multiplier design for ECG signal processing, built on quantum technology. This innovative approach merges Vedic mathematics with quantum-based nano-architectures to create a highly efficient, low-power arithmetic unit capable of handling real-time biomedical data. The design is particularly aimed at accelerating signal processing tasks in medical applications, achieving higher computational speed, reduced latency, and energy efficiency all crucial for next-generation healthcare technologies^30^. conducts an in-depth investigation into autonomous learning techniques for complex pattern recognition within interconnected information systems. It examines advanced machine learning approaches, particularly deep learning models that enable systems to self-learn and adapt to evolving data patterns without extensive human intervention. The study emphasizes scalability, real-time responsiveness, and accuracy, making it highly relevant for applications in dynamic and data-intensive environments^31^. introduce an innovative performance assessment method for improving the efficiency of the Ad hoc On-Demand Distance Vector (AODV) routing protocol in Vehicular Ad Hoc Networks (VANETs) using Coloured Timed Petri Nets (CTPNs). This approach enables a more precise and formal modelling of protocol behaviour under dynamic and time-sensitive conditions, allowing for thorough performance evaluation and optimization of routing mechanisms in highly mobile and latency-critical environments.

**Table 1:** Overview of key studies and their relevance to secure and efficient federated learning in telesurgery.

Reference no	Year	Key technique/algorithm	Contribution summary	Relevance to telesurgery
^20^	2024	Grey Wolf Optimization + MapReduce	Efficient service composition and load balancing	Enhances scalability and execution speed in decentralized healthcare systems
^21^	2024	Glowworm Swarm Optimization (GSO)	Multi-objective optimization balancing security, latency, and throughput	Relevant for secure and responsive medical device networks
^22^	2025	Blockchain + Deep Reinforcement Learning (DRL)	Trustworthy, low-latency data offloading framework	Directly supports federated learning in telesurgery with secure data handling
^23^	2024	Fault-tolerant Modelling	Reliability analysis under permanent fault conditions	Improves reliability evaluation of telesurgical sensor networks
^24^	2024	Nature-Inspired Algorithms	Systematic review of metaheuristics in healthcare services	Highlights optimization strategies for data and decision-making in medical systems
^25^	2024	GSO	Improves latency, throughput, and security	Addresses critical QoS trade-offs for telesurgical blockchains
^26^	2024	Survey of Botnet Threats	Review of botnet evolution and defense mechanisms	Useful for enhancing telesurgery security architecture
^29^	2025	Multi-core + GPU Parallelism	High-speed real-time simulation	Inspires GPU use in telesurgery for low-latency data/image processing
^28^	2025	Quantum Vedic Multiplier	High-speed, low-power arithmetic design	Supports real-time biosignal analysis in telesurgery devices
^30^	2024	Deep Learning	Autonomous learning for complex data	Enhances adaptive learning in dynamic telesurgical environments
^31^	2025	Colored Timed Petri Nets (CTPNs)	Formal performance evaluation of AODV protocol	Applicable to modeling and optimizing telesurgery network protocols
^27^	2025	Quantum ALU Design	Nano-scale ALU for efficient computing	Boosts processing efficiency in edge-based telesurgery
^28^	2025	Quantum Vedic Multiplier	Quantum-enhanced ECG processing unit	Reinforces energy-efficient, fast edge analytics for surgical sensors

To advance toward highly secure and privacy-preserving tele-surgery solutions, innovative methods, frameworks, protocols, and validation techniques must be developed while minimizing security and privacy trade-offs. However, existing systems exhibit limitations in terms of privacy protection, reliability, cost-effectiveness, and data processing efficiency. Additionally, they remain vulnerable to various security threats, including modification attacks, spoofing, and other cyber risks that could compromise the integrity of remote surgical procedures. In recent years, the convergence of AI, 5G/6G wireless communication, IoT, and edge computing has begun reshaping the landscape of healthcare, particularly in the domain of robotic surgery. The integration of these technologies is driving significant improvements in precision, speed, and real-time decision-making for clinicians. This transformation is best exemplified by advancements in tele-surgery and tele-mentoring, where enhanced situational awareness and real-time feedback are vital for successful outcomes.

### Motivation

One of the critical benefits of this convergence is latency reduction, which is essential for real-time, remote surgical procedures. For instance, the integration of 5G networks has demonstrated the potential to reduce latency significantly, enabling near-instantaneous communication between surgical teams and robotic systems. A study by^32^ discusses the low-latency, high-throughput characteristics of 5G that make it viable for robotic telesurgery, where every millisecond of delay can impact surgical precision. The deployment of edge computing further complements this by processing data closer to the source, eliminating the need for extensive data transmission to central servers. This reduces delays and improves data throughput, which is essential for handling the vast amounts of sensory data generated by robotic surgical tools^33^. highlight how mobile edge computing in healthcare systems reduces latency and enhances real-time decision-making by enabling localized processing of IoT-enabled devices. Moreover, the incorporation of AI algorithms in robotic surgery is improving surgical precision by providing real-time analysis of patient data. This AI-enhanced capability helps surgeons make informed decisions on the fly, reducing the risk of human error. The use of AI and IoT devices to facilitate personalized treatment and automated monitoring is revolutionizing healthcare^34^. describe how AI combined with edge computing improves patient monitoring systems by enabling real-time feedback to clinicians, which is critical during complex surgeries. In terms of surgical outcomes, the convergence of these technologies is leading to improvements in operational efficiency and patient care. Smart hospitals equipped with 5G-enabled robotic systems, for example, offer more efficient and precise surgical operations. This is confirmed in the study^35^, which presents the case of a 5G-enabled smart hospital where AI-driven tools work in tandem with IoT devices to enhance surgical precision and patient safety. In a telesurgical setting, sensitive patient data, including high-resolution video streams, audio commands, and real-time control signals, must traverse public networks. This opens the system to a range of cyber threats such as spoofing, data injection, eavesdropping, and model poisoning. Moreover, centralized data processing models raise significant privacy concerns and become bottlenecks under adversarial conditions. Current solutions lack the scalability, adaptability, and layered security required for the real-world implementation of secure telesurgical systems. Privacy and interpretability are fundamental in telesurgical systems, especially when biometric data is used for authentication or decision support. Traditional black-box models often fall short in healthcare applications due to their lack of transparency, which can hinder clinical trust. Addressing this concern^36^, introduced a framework that combines explainable artificial intelligence (XAI) with synthetic biometric data processing. Their approach ensures that sensitive biometric patterns are processed in a privacy-preserving manner while providing transparent, interpretable insights into decisions. This dual focus on data anonymization through synthetic generation and model explainability is highly relevant to telesurgery, where real-time decisions must be both secure and justifiable. Incorporating such techniques into telesurgical platforms can improve compliance with privacy regulations and increase trust among practitioners and patients alike.

### Problem statement

1. To address the increased risk of cyberattacks in cloud-based telesurgical systems.
2. To overcome privacy concerns associated with centralized data aggregation.
3. To introduce a lightweight yet highly accurate model for real-time multi-attack detection.
4. To ensure resilience and scalability for next-generation medical IoT systems.

To address these critical gaps, this research proposes a Federated Optimized Gated Transformer Learning (FOGTL) framework tailored for cloud-based telesurgical environments. Our approach leverages Federated learning (FL) to preserve patient data privacy by enabling decentralized training, while integrating swarm intelligence-based optimization and chaotic encryption for robust security and model efficiency.

### Core contributions

This work contributes to the ongoing effort to enable secure, decentralized, and intelligent telesurgical systems using advanced machine learning techniques and aligns with current research priorities in secure AI deployment in healthcare.

- This study suggests integrating to develop a novel federated learning framework integrated with chaotic encryption to ensure secure communication and model aggregation in telesurgical environments.
- To design an OGTN, enhanced using the Stadium Spectator Optimization (SSO) algorithm, to improve anomaly detection accuracy and responsiveness, and to apply federated learning to enable decentralized, privacy-preserving model training across distributed medical devices without sharing raw patient data.
- Finally, to validate the proposed framework using the UNSW-NB15 benchmark dataset and demonstrate superior performance over state-of-the-art GTN-based models, and to conduct statistical security analysis using NIST tests to verify the robustness of the chaotic encryption module against adversarial threats.

The rest of the paper is structured as follows: Section II provides a concise overview of current FL approaches designed for IoT and WBAN-IoT, highlighting their resilience against various security threats. The preliminaries are demonstrated in Section III. Section IV elaborates on the stages of the proposed framework, which is implemented within teleoperated surgical systems to enhance efficiency and security. Section V represents the experimental analysis, theoretical insights, and detailed comparative assessments, demonstrating the effectiveness of the recommended approach. Finally, Section VI wraps up the key findings of this research and outlines potential future improvements to further refine and optimize the framework.

## Related works

^37^ presented an FL-driven Cybersecurity Framework for IoT environments that uses recurrent neural networks (RNNs) for anomaly detection. The framework implements local model training on edge devices, where each device processes its data independently, followed by secure model aggregation using homomorphic encryption to protect sensitive information during the learning process. The system achieved over 98% accuracy in detecting complex cyber threats like DDoS attacks and demonstrated a 20% cut in resource utilization when compared to centralized models. However, the study doesn’t address model performance across diverse IoT device types^38^. develop a comprehensive cloud security model that synergizes FL using reinforcement learning, decision trees, and Zero Trust Network Access principles. Their empirical evaluation showed notable improvements in response to delay. While the results are promising, the study doesn’t thoroughly examine the computational overhead of implementing multiple advanced technologies simultaneously or address potential integration challenges in legacy cloud systems^39^. represented a novel SCNN-Bi-LSTM scheme for intrusion recognition in wireless sensor networks, combining Stacked Convolutional Neural Networks (CNN) with Bidirectional Long Short-Term Memory (Bi-LSTM) networks in a federated learning framework. Testing on WSN-DS and CIC-IDS-2017 datasets, their approach achieved remarkable classification. Architecture enables the detection of various types of DoS attacks while preserving user data privacy. A key limitation is the lack of evaluation under resource-constrained conditions typical in real-world WSN deployments^40^. DL techniques offer a viable way to improve IoT network security and lower security threats by efficiently identifying anomalies in network data. To defend IoT networks against cyberattacks, this study proposes a DL-based IDS that uses Feed-Forward Neural Networks (FFNN), Long Short-Term Memory (LSTM), and Random Neural Networks (RandNN).

^41^ developed an FL framework for DDoS threat recognition in IoT networks. Using the N-BaIoT dataset, their study demonstrates that FedAvgM outperforms FedAvg for non-IID data distributions. While the research addresses non-IID challenges through retraining and partial selection techniques, it doesn’t extensively evaluate the system’s performance under varying network criteria or explore the impact of stragglers on model convergence^42^. evaluate optimization approaches for client selection in FL cybersecurity applications, including (GWO), Particle Swarm Optimization (PSO), and Cuckoo Search (CS), across scenarios of fixed/dynamic participation, non-IID data, and adversarial conditions. Their research demonstrates that Grey Wolf Optimization achieves superior performance in accuracy, recall, and F1 scores across all configurations. However, the study doesn’t provide a detailed analysis of the computational complexity trade-offs between different SI algorithms or examine their scalability for large-scale deployments^43^. proposed a framework combining FL with blockchain technology for IoT security using the N-BaIoT Dataset. They evaluated two approaches: Logistic Regression, achieving 99.98% global, and Dense Neural Networks, reaching 99.99% accuracy. The study introduces novel metrics, the Security Efficacy Metric and Comparative Improvement Factor, for framework evaluation. However, the research doesn’t address the scalability challenges of blockchain integration in resource-constrained IoT devices^44^. explored cloud computing security enhancement by Deep Belief Networks (DBN) for intrusion recognition. Their Deep Belief Networks approach achieved 94.4% detection accuracy with a 2.2% false positive rate and 158.4ms computational efficiency, while the Attention Mechanism showed superior results with 96.2% accuracy, 1.1% false positives, and 153.2ms efficiency. The study lacks an analysis of the system’s performance under varying network loads and attack patterns^45^. developed a security model using elevated principal component analysis (IPCA) for feature extraction and a hybrid grasshopper-crow search optimization (GSCSO) for feature selection, coupled with an isolated heuristic neural network (IHNN) for prediction. While effective, the study doesn’t address the real-time implementation challenges in production environments^46^. presented a federated learning approach for IoT security using GRUs and an ensembler for model aggregation. Their method maintains data privacy by keeping information on local devices and sharing only learned weights with the central server. The approach demonstrated superior performance compared to centralized machine learning methods in both privacy preservation and attack detection accuracy. However, the research doesn’t fully explore the communication overhead among devices and the central server^47^. provided a theoretical analysis of federated learning integration in edge and cloud computing for cybersecurity. The study examines the benefits in privacy-preserving data analysis and real-time threat recognition while identifying challenges like model poisoning and communication overhead. Though comprehensive in theoretical analysis, the paper lacks empirical validation of the proposed concepts and specific implementation guidelines. According to^48^, to protect IoT devices from a variety of threats, especially Distributed Denial of Service (DDoS) attacks, intrusion detection systems (IDS) are essential. Since these attacks have grown to be a serious threat to IoT networks, sophisticated approaches to decision-making are necessary to mitigate the serious hazards they pose^49^. demonstrated how a computational blockchain process with offloading analysis can enhance security in federated learning systems by incorporating blockchain-based mechanisms for secure aggregation^50^. proposed a lightweight blockchain model that combines artificial intelligence with blockchain technology to secure the federated learning process in IIoT systems, providing both security and privacy. In healthcare applications, where real-time predictions are crucial, optimization methods like those proposed by^51^ ensure that the federated learning system remains efficient and privacy-preserving while maintaining high prediction accuracy^52^. highlighted the use of GANs in the detection of fraud in evolving systems, where adversarial networks can be trained to identify fraudulent behavior in real-time^53^. demonstrated how synthetic healthcare data can be generated using biometric patterns for use in federated learning systems, enhancing both data privacy and model accuracy. For secure federated learning implementations in IoT environments, utilizing advanced intrusion detection systems and optimization algorithms is essential. Techniques such as Decisive Red Fox optimization can be used for improving the detection accuracy and reducing false positives in IoT systems^54^. Recent work by^55^ introduced SPARK and SAD, two leading-edge deep learning frameworks for robust and effective intrusion detection in SCADA systems, which could be adapted to federated learning frameworks in critical infrastructure. These frameworks utilize advanced deep learning techniques to detect and mitigate threats in real-time, ensuring that both local and global models remain secure during training. Adapting these methodologies could significantly enhance the security of federated learning systems which shown in Table 2, particularly in scenarios where model updates must be protected from adversarial attacks or data poisoning.

**Table 2:** Summary of related work highlighting author contributions, methodologies, strengths, and limitations.

Reference(s)	Contribution	Methods/techniques used	Strengths	Limitations
^25^	FL framework for IoT cybersecurity	RNNs, homomorphic encryption	98%+ accuracy on DDoS; 20% resource savings	No performance analysis across heterogeneous IoT devices
^26^	Cloud security model with FL, RL, and Zero Trust principles	Reinforcement learning, decision trees	Reduced response delay	Lacks computational overhead and legacy integration assessment
^29^	SCNN-Bi-LSTM model for intrusion detection in WSNs	CNN + Bi-LSTM in FL	High accuracy on WSN-DS and CIC-IDS-2017; maintains privacy	Not evaluated under resource-constrained WSN conditions
^40^	DL-based IDS for anomaly detection in IoT	FFNN, LSTM, RandNN	Effective threat detection	Lacks federated or performance-specific insights
^28^	FL-based DDoS detection in IoT using N-BaIoT	FedAvgM, partial retraining	Better non-IID handling with FedAvgM	No evaluation of performance under network variations or stragglers
^30^	Optimized client selection for FL in cybersecurity	GWO, PSO, CS algorithms	GWO outperforms others in accuracy and recall	No detailed complexity/scalability analysis
^31^	Blockchain-integrated FL for IoT security	Logistic Regression, Dense NN, blockchain	99.98–99.99% accuracy; introduces new security metrics	Scalability concerns with blockchain in low-power devices
^27^	Cloud security enhancement with DBN and Attention Mechanism	Deep Belief Networks, Attention	Up to 96.2% accuracy; low false positives and high efficiency	No test under diverse network loads and attack types
^28^	Secure prediction using optimized feature selection + IHNN	IPCA, GSCSO, IHNN	Efficient prediction pipeline	No discussion of deployment in real-time environments
^32^	FL framework using GRUs and ensemble for IoT intrusion detection	GRU, model ensembling	High privacy preservation; strong accuracy	Communication overhead between clients and server not detailed
^33^	Theoretical study on FL in edge/cloud cybersecurity	Conceptual FL integration	Insightful privacy and threat recognition analysis	No empirical experiments or deployment strategies
^34^	Importance of IDS for mitigating DDoS attacks in IoT	Intrusion Detection Systems (general)	Highlights need for advanced IDS in IoT	General discussion; lacks technical or architectural proposals

## Preliminaries

### Gated recurrent units

GRUs are a streamlined variant of recurrent neural networks (RNNs), designed to capture temporal dependencies in sequential data with lower computational complexity than traditional Long Short-Term Memory (LSTM) networks. This makes GRUs particularly suitable for real-time and resource-constrained applications such as telesurgery, where timely signal processing is critical. Each GRU cell utilizes two key mechanisms:

- The *update gate*, which controls the extent to which past information is preserved.
- The *reset gate*, which determines the influence of prior hidden states on the current input.

These gates collaboratively regulate the flow of information through time, enabling the GRU to selectively remember or discard parts of the input history. This dynamic control allows the GRU to extract context-aware features from encrypted telemetry or patient data. In our framework, GRUs are employed to analyze time-series data streams (e.g., network traffic, biosensor signals) and extract deep sequential features. The final hidden state of the GRU encapsulates the temporal behaviour of the input, serving as a condensed representation for further classification or anomaly detection modules.

### Gated transformer network (GTN)

It is an advanced neural network architecture that builds upon the Transformer model by incorporating a gating mechanism to selectively filter and prioritize information during sequence processing. This gating mechanism enhances the self-attention mechanism of the Transformer, allowing the model to dynamically focus on the most relevant features of the input sequence while discarding less important data. GTNs combine the strengths of the Transformer’s ability to capture long-range dependencies with the adaptability of the gating mechanism, improving the model’s efficiency and performance in tasks such as natural language processing (NLP), time-series forecasting, and speech recognition. The architecture is particularly beneficial for tasks involving sequential data, as it can more effectively handle redundancy and emphasize critical information, making it a powerful tool for a wide range of applications. GRUs are efficient for sequential data processing, while GTNs, with their self-attention mechanism, excel in parallel processing and handling long-range dependencies in sequences. The Table 3 summarizes the key differences between GRUs and GTNs in terms of architecture, data processing, efficiency, and complexity.

**Table 3:** Key differences between GRUs and GTNs.

Feature	GRU	GTN
Architecture	Part of recurrent neural networks (RNNs)	Part of the transformer architecture with a gating mechanism
Data processing	Processes data sequentially, one time step at a time	Processes entire sequence in parallel using self-attention
Gating mechanism	Uses reset and update gates to control information flow	Uses gating in the self-attention mechanism to focus on relevant information
Long-range dependency handling	Struggles with long-range dependencies due to vanishing gradients	Efficiently captures long-range dependencies via self-attention
Computational efficiency	Slower for long sequences due to sequential processing	Faster for long sequences as it processes data in parallel
Complexity	Simpler architecture with fewer parameters	More complex with greater computational requirements
Application	Suitable for sequential data with shorter-term dependencies	Suitable for tasks requiring long-range dependencies and parallel processing

GTNs are preferred over GRUs in telesurgery due to their ability to handle complex, real-time tasks efficiently. Unlike GRUs, which process data sequentially, GTNs leverage parallel processing, enabling them to handle large volumes of data in real time, crucial for telesurgery where low latency is essential. GTNs excel at capturing long-range dependencies through their self-attention mechanism, making them better suited for tasks that require understanding relationships between distant data points, such as in surgical tool movements or patient vitals. Additionally, GTNs are more adept at managing noisy and unstructured data, as their attention mechanism allows them to focus on relevant features, while GRUs may struggle with noisy sequences. Their scalability also makes them ideal for multi-modal learning, where different data types (e.g., sensor data, video, haptic feedback) must be processed together. Furthermore, GTNs offer improved performance in complex contexts, better generalization, and transfer learning, which are valuable in dynamic telesurgical environments. Overall, GTNs’ parallel processing, ability to handle long-range dependencies, multi-modal data integration, and low-latency performance make them a more suitable choice for enhancing the effectiveness and safety of telesurgery.

*Chaotic gated transformer (CGT)*: It is a novel variant of the Gated Transformer, designed to harness the advantages of chaotic dynamics within the self-attention mechanism. By incorporating chaotic systems into the gating process, we aim to improve the model’s ability to handle complex patterns, explore non-linear relationships in the data, and enhance its robustness to noise, especially in scenarios where data distributions are unpredictable or highly variable. The core idea behind CGT is to integrate chaotic processes into the attention mechanism in a controlled way, leveraging their ability to explore multiple configurations of attention weights and escape local optima during training. This results in a more flexible and adaptive model, capable of addressing problems in highly dynamic environments, such as federated learning and real-time decision-making systems. The CG.

T builds on the multi-head self-attention mechanism from standard Transformer models. In each attention head, the chaotic gating process modulates the attention weights, allowing for more flexible and dynamic learning of contextual relationships. The chaotic system injects variability in the attention pattern, which can be particularly beneficial for tasks where patterns are not deterministic. The Table 4 which describes the variation between standard, gated and chaotic gated transformer is shown below:

**Table 4:** Comparison of standard, gated, and chaotic gated transformers across key architectural and performance features.

Feature	Standard transformer	Gated transformer	Chaotic gated transformer
Attention mechanism	Pure self-attention	Gated self-attention	Gated + chaos-modulated attention
Control of information	No gates	Learnable gates	Gates modulated by chaotic systems
Handling non-linearity	Moderate	Better than standard	High (via chaos)
Adaptability	Static	Dynamic gating	Dynamically chaotic + gated
Robustness to noise	Limited	Moderate	Strong
Optimization landscape	Deterministic	Semi-flexible	Chaotic exploration (avoids local minima)
Suitable for	Structured, clean data	Tasks with partial noise	Noisy, non-linear, distributed learning

### Federated learning in telesurgery

Federated learning (FL) offers several key advantages in securing distributed robotic surgery systems, primarily by addressing the risks associated with centralized data processing and minimizing the potential attack vectors. The decentralized nature of FL enhances both privacy and security in the context of telesurgery:

i. *Prevention of Data Exfiltration*: By design, federated learning ensures that patient data never leaves the local device. Instead of sending sensitive patient records or surgical data to a central server, only aggregated model parameters (such as weights and gradients) are shared between participating nodes. This approach minimizes the risks of data exfiltration, which could otherwise lead to data breaches or unauthorized access to confidential medical information. As a result, federated learning supports the privacy-preserving nature of telesurgery systems, where patient data remains secure across institutions^56^.
ii. *Enhancement of Adversarial Robustness*: Federated learning improves the robustness of the model against adversarial attacks, such as data poisoning and model manipulation. In traditional centralized learning, adversaries may manipulate the central model by injecting malicious data into the system. In contrast, federated learning aggregates the knowledge of multiple decentralized nodes, making it harder for adversaries to poison the model without compromising multiple participants. Even if one node is compromised, it does not significantly degrade the performance or accuracy of the global model, thereby enhancing adversarial robustness. This is particularly crucial in robotic surgery, where model reliability and precision are paramount for patient safety^56^.
iii. *Reduction of Central Server Dependency*: Federated learning reduces the dependency on a central server by allowing distributed nodes to perform local model training and only share model updates. This architectural choice significantly mitigates the risk of centralized server-based attacks, such as Denial-of-Service (DoS) or Man-in-the-Middle (MITM) attacks. In a traditional model, the compromise of a central server could lead to large-scale system failures. However, with federated learning, even if an attacker compromises one of the nodes, the damage is contained, and the system remains operational as other nodes continue learning independently. This distributed resilience improves the overall security and availability of the robotic surgery systems^57^.

###  Layered, optimized chaotic encryption in federated learning

To secure data in the federated learning process, we employ layered, optimized chaotic encryption schemes based on chaotic maps (logistic and tent maps). These maps generate pseudo-random sequences that act as encryption keys, ensuring data confidentiality and preventing unauthorized access to sensitive information during model training. The encryption is applied in multiple layers to enhance security, and synchronization across federated nodes is achieved by securely sharing initialization seeds. Through this approach, we ensure that the encryption process introduces minimal latency, thus preserving the real-time performance required for robotic surgery applications.

### Cybersecurity threats in telesurgery

The adoption of telesurgery is hindered by several unforeseen cyber threats that pose significant risks to the security, privacy, and integrity of both the robotic systems and the data transmitted during surgical procedures. These threats must be addressed to ensure the safety and reliability of teleoperated healthcare systems. The key threats identified in recent research include:

*Adversarial model poisoning:* Adversarial attacks targeting machine learning models used in robotic surgery systems can lead to compromised decision-making. In federated learning systems, which are often employed to enable collaborative model training across multiple institutions, adversaries can inject malicious data into the training sets. These poisoned datasets can alter the model’s behavior, resulting in erroneous or unsafe surgical actions. Such attacks are particularly challenging because they may go undetected during training but can significantly impact the model’s reliability in real-time surgical scenarios^58^.

*Man-in-the-Middle (MITM) attacks:* MITM attacks are a significant concern in telesurgery, as they allow attackers to intercept and manipulate the communication between the surgeon and the robotic system. During a surgery, real-time data such as vital signs, imaging, and control commands are transmitted over networks. If attackers gain access to this communication stream, they could inject false data or manipulate the commands, leading to disastrous outcomes. Recent studies on robotic surgery systems highlight the importance of securing communication channels to prevent MITM attacks, which could compromise the safety of patients^59^.

*Real-time data leakage:* Real-time data leakage is another critical threat that arises in telesurgery, especially when using IoT devices for remote monitoring and control. Sensitive patient information such as medical history, imaging data, and live surgical footage is often transmitted over networks, making it vulnerable to unauthorized access. If data encryption is not properly implemented, attackers may intercept or leak this information, violating patient privacy and potentially leading to financial or reputational damage to healthcare institutions. Ensuring secure data transmission through robust encryption and authentication protocols is essential^60^.

*Denial-of-service (DoS) attacks:* DoS attacks, where malicious actors overwhelm the system with excessive traffic, can disrupt the operation of telesurgery systems by preventing critical communication between the surgeon and the robotic arm. Such disruptions could delay the operation or even halt it altogether, endangering the patient’s life. DoS attacks are particularly concerning in real-time medical procedures where every second counts. Research on network resilience and attack mitigation in robotic surgery systems emphasizes the need for robust defences to ensure uninterrupted communication during surgeries^61^.

*Ransomware attacks:* Healthcare systems, including telesurgery platforms, are increasingly targeted by ransomware attacks. In these attacks, hackers encrypt critical system data and demand a ransom to restore access. If ransomware targets the robotic surgery system or the communication infrastructure, it can disrupt surgery and render essential medical data inaccessible. Ransomware attacks on healthcare institutions are rising, and their impact on telesurgery systems could be catastrophic if not properly safeguarded^62^.

## Proposed methodology

The proposed framework comprises three major components: (i) a Gated Recurrent Unit (GRU) network optimized for attack classification, (ii) a custom hyperparameter tuning strategy based on Stadium Spectator Optimization (SSO), and (iii) secure model update aggregation via Federated Learning (FL), enhanced with the Optimistic Chaotic Encryption Layer (OCEL) for privacy preservation. Each component is elaborated in detail below.

### Materials and methods

The UNSW-NB15 dataset, initially introduced in^63^, provides a more realistic and contemporary synthetic network traffic dataset compared to previous Network Intrusion Detection System (NIDS) datasets like KDD99^64^, , and NSL-KDD^65^. This dataset contains approximately 2.5 million records, categorized into one normal class and nine attack types: Backdoor, Analysis, Exploits, Denial-of-Service (DoS), Generic, Fuzzers, Reconnaissance, Worms, and Shellcode. In this study, we utilized the UNSW-NB15 dataset, which is widely recognized for evaluating intrusion detection and cybersecurity models. This dataset was generated using the IXIA Perfect Storm tool in a cyber range lab at the Australian Centre for Cyber Security (ACCS). It captures a wide range of normal and malicious network activities, making it suitable for modelling threat detection in tele-surgical systems where data privacy and security are of utmost importance.

#### Dataset composition and class distribution

The UNSW-NB15 dataset consists of a mixture of normal traffic and nine distinct types of malicious traffic, which include DoS (Denial of Service), Fuzzers, Generic attacks, Exploits, Reconnaissance, Backdoor, Analysis, Shellcode, and Worms. A critical aspect of this dataset is its high-class imbalance, which directly affects the performance and fairness of machine learning models. The “Normal” class dominates the dataset, accounting for approximately 87% of the total records. The DoS class, which represents an overwhelming volume of service requests meant to disrupt systems, contributes about 8% of the total. Other attack types, such as Fuzzers, Generic, Exploits, and Reconnaissance, make up approximately 1–2% each, while Backdoor, Analysis, ShellCode, and Worms each constitute less than 1%. This skewed distribution presents a real-world scenario where malicious events are rare but critical, reinforcing the need for robust classification techniques that can handle class imbalance without biasing towards majority classes.

#### Data preprocessing and splitting strategy

**Fig. 1:**
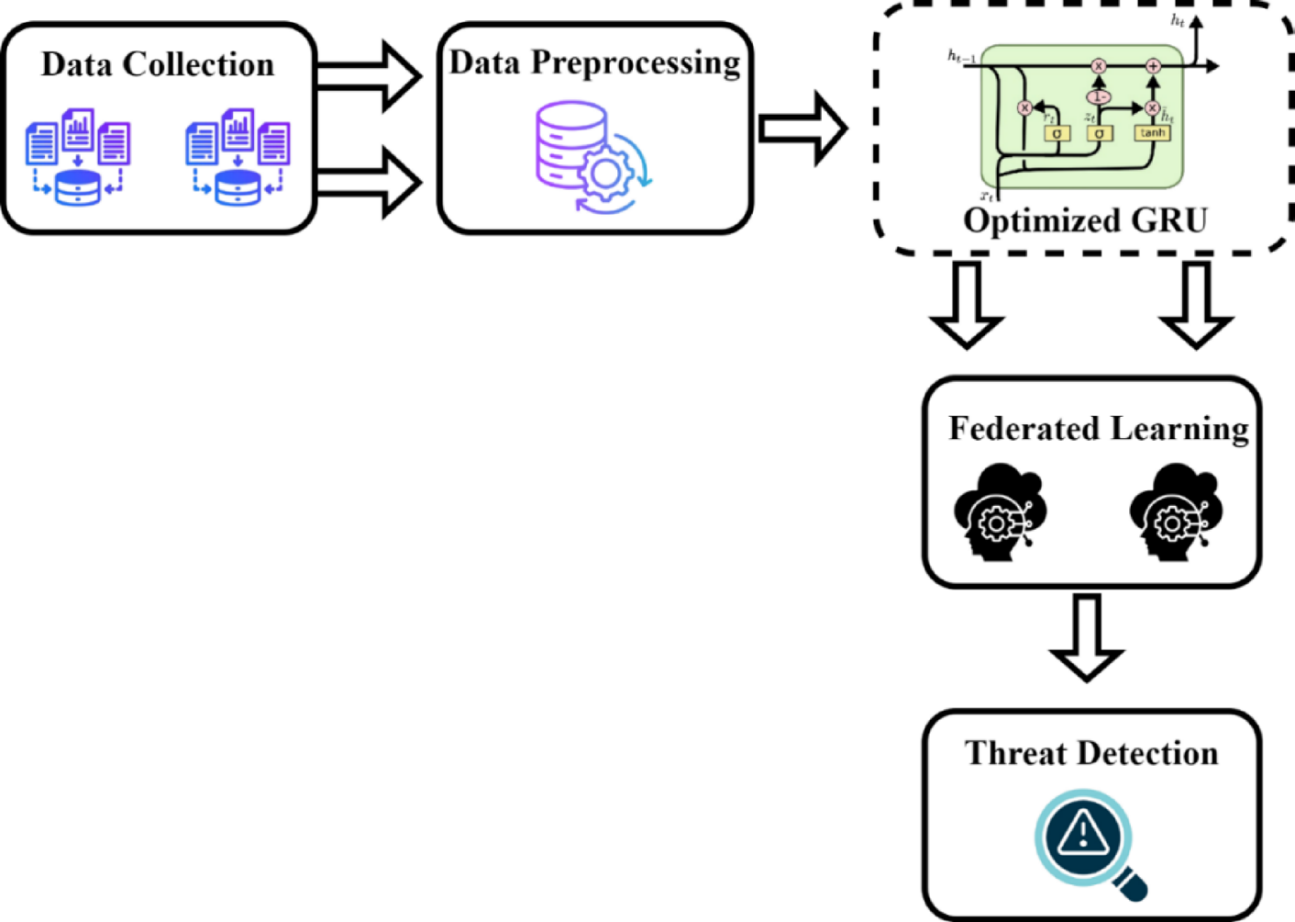
Proposed architecture of the federated learning-based telesurgery system.

To train and evaluate the proposed Federated Optimized Gated Transformer Learning (FOGTL) framework which is shown in Fig. 1, the dataset was divided into training and testing subsets using a 68:32 ratio. Specifically, 68% of the dataset was used to train the model, allowing it to learn complex patterns, relationships, and anomalies in the network traffic. The remaining 32% served as a test set to validate the model’s predictive performance on unseen data, thus ensuring the model’s ability to generalize. This splitting ratio balances the need for sufficient training samples while maintaining a reliable test set for objective evaluation. The preprocessing stage also included normalization of numerical features, encoding of categorical features, and shuffling of the dataset to prevent ordering bias. Min-max normalization is the technique employed, applying a linear transformation. This pre-processing produces new normalized dataset versions from the original raw data. After pre-processing, One-hot encoding is deployed in the datasets to convert all the string values into numeric values.

In addition, K-fold cross-validation (with K = N) was applied to ensure robust evaluation. In each fold, the model was trained on (K-1) subsets and validated on the remaining subset. This process was repeated N times, and the final performance metrics reflect the average results across all folds, minimizing overfitting and providing a more reliable performance estimate. The preprocessing phase included min-max normalization for numerical features, one-hot encoding for categorical data, and shuffling to avoid ordering bias. These transformations ensured that all input data was in a standardized, model-friendly format before training.

### Hyperparameter optimization using stadium spectator optimization (SSO) model

The Stadium Spectator Optimization (SSO) algorithm is a population-based metaheuristic inspired by dynamic interactions within a crowded stadium. While the original conceptualization used spectators and players as metaphors, this section focuses on a technically grounded formulation of the algorithmic processes involved. SSO begins with a population of candidate solutions, each representing a potential answer to the given optimization problem. These candidates are distributed across a multidimensional search space defined by the problem’s decision variables. The algorithm evolves the population over successive iterations through stochastic operators guided by behavioral principles, such as influence, reaction, and adjustment. Each candidate’s position is updated based on three core influences:

1. *Peer Influence (Exploration)*: Randomly selected candidate solutions are used to explore distant regions of the search space. This mechanism ensures diversity and helps the algorithm avoid premature convergence.
2. *Elite Influence (Exploitation)*: The best solution found so far guides other candidates toward promising regions. This local search mechanism intensifies the search around high-quality solutions.
3. *Stochastic Adaptation*: Random vectors (uniform and normal distributions) introduce perturbations in candidate positions, encouraging nonlinear movement through the search space. This randomness emulates unpredictable factors and introduces a balance between exploration and exploitation.

A control parameter dynamically adjusts the emphasis between exploration and exploitation based on the current iteration count. Early in the process, exploration is favored to maximize coverage, while later iterations emphasize exploitation to fine-tune high-performing candidates. To ensure feasibility, the algorithm applies boundary control. Any candidate solution that violates the predefined bounds of the problem’s variables is corrected and projected back into the allowable region. The update rule integrates multiple randomly generated factors and elite guidance to recalibrate each candidate’s position. This hybrid mechanism ensures both global search capability and local refinement, making the SSO effective for complex, nonlinear optimization tasks. The algorithm continues until a termination condition is met typically a fixed number of iterations or convergence criteria. The final output is the best candidate solution encountered during the optimization process^40^.

#### Mathematical model

The computational formulation of the Spectator–Spectacle Optimization (SSO) approach is introduced in this segment. The process initiates with the initialization of candidate solutions, denoted as X_i_, which represent the reactions of players in the stadium to the actions of spectators. This relationship can be computationally depicted as follows:

1 $$\:X=\left[\begin{array}{c}{X}_{1}\\\:{X}_{2}\\\:\vdots\\\:{X}_{i}\\\:\vdots\\\:{X}_{n}\end{array}\right]=\left[\begin{array}{cccccc}{x}_{1}^{1}&\:{x}_{1}^{2}&\:\dots\:&\:{x}_{1}^{j}&\:\dots\:&\:{x}_{1}^{d}\\\:{x}_{2}^{1}&\:{x}_{2}^{2}&\:\dots\:&\:{x}_{2}^{j}&\:\dots\:&\:{x}_{2}^{d}\\\:\vdots&\:\vdots&\:\vdots&\:\vdots&\:\ddots\:&\:\vdots\\\:{x}_{i}^{1}&\:{x}_{i}^{2}&\:\dots\:&\:{x}_{i}^{j}&\:\dots\:&\:{x}_{i}^{d}\\\:\vdots&\:\vdots&\:\vdots&\:\vdots&\:\ddots\:&\:\vdots\\\:{x}_{n}^{1}&\:{x}_{n}^{2}&\:\dots\:&\:{x}_{n}^{j}&\:\dots\:&\:{x}_{n}^{d}\end{array}\right]\:;\:\left\{\begin{array}{c}i=\text{1,2},\dots\:,n.\\\:j=\text{1,2},\dots\:,d.\end{array} \right.$$

2 $$\:{x}_{i}^{j}={x}_{i,min}^{j}+rand.\left({x}_{i,max}^{j}-{x}_{i,min}^{j}\right)\:;\: \left \{\begin{array}{c}i=\text{1,2},\dots\:,n.\\\:j=\text{1,2},\dots\:,d.\end{array} \right.$$.

In the SSO algorithm, n denotes the entire count of candidate solutions, while dd signifies the dimensionality of the optimization problem. The variable $\:{x}_{i}^{j}$ represents the jth decision variable corresponding to the initial position of the ith candidate. The terms $\:{x}_{i,min}^{j}$ and $\:{x}_{i,max}^{j}\:$define the lower and upper boundaries for the jth variable in the ith candidate. Additionally, “*rand*” refers to a uniformly distributed random value ranging between zero and one.

The computational formulation is given in Eqs. (1) and (2) and is utilized to initialize search agents within the optimization problem’s search space. These agents influence player responses, leading to variations in the objective function values. Throughout the loop’s execution of the approach, each candidate’s solution$\:{\:X}_{i}$ is updated with new values to refine the population, thereby enabling efficient exploration and exploitation across varied areas of the search space.

The two search agents are selected arbitrarily over the population to execute a search approach in every iteration within the SSO technique. The approach also incorporates the search agent associated with the optimal solution identified so far to further guide the search process. A randomly generated value within the range of zero to one dictate whether the algorithm prioritizes exploration or exploitation.

For exploitation, the algorithm shifts from the best-known solution to a location within the region among the two randomly chosen search agents, producing two alternative solutions to the optimization problem. In contrast, the search agent moves beyond the best answer via the exploration phase, jumping into a different location through the search space. This transition results in variations in the players’ responses to spectators’ actions, ultimately affecting the optimization outcome. The values derived from both exploration and exploitation behaviors are stored in a vector termed RND, which is mathematically formulated as follows:

3 $$\:\overrightarrow{RN{D}_{i}}=\{\begin{array}{c}\overrightarrow{{\text{XB}}_{i}}+\overrightarrow{({X}_{R1}}-\overrightarrow{{X}_{R2})\:\:\:;\:\:}ifHR\le\:0.5\\\:\overrightarrow{{\text{XB}}_{i}}+\overrightarrow{R}\:\:\:\:\:\:\:\:\:;\:\:otherwise\end{array}$$.

In Eq. 3, $\:\overrightarrow{{\text{XB}}_{i}}\:$represents the best solution identified up to the current iteration, while $\:\overrightarrow{{X}_{R1}}\:$and $\:\overrightarrow{{X}_{R2}}\:$denote two randomly selected search agents. ‘RND’ is the vector made up of equally distributed integers that are arbitrarily created inside the same interval as the variable ‘*HR*’, which is an arbitrary number between 0 and 1. A control parameter has been implemented in order to reconcile the exploration and exploitation phases of the SSO technique and efficiently govern the maneuvering of search agents through the technique. This parameter ensures the appropriate trade-off between diversification (exploration of new regions in the search space) and intensification (refinement of promising solutions). The computational formulations of this control parameter are given as follows:

4 $$\:LI=1-(\text{I}/\text{Im})$$.

In Eq. 4, ‘I’ represent the present iteration count, while ‘*Im*’ signifies the total number of iterations allowed in the SSO algorithm. Taking these factors into account, the process of updating the candidate solutions at each iteration follows the subsequent mathematical formulation is shown in Eq. 5:

5 $$\:\overrightarrow{NewPositio{n}_{i}}=\overrightarrow{{\text{X}}_{i}}+\overrightarrow{r1}\cdot\:LI\cdot\:\left(mu1\otimes\:\overrightarrow{r2}\cdot\:\left(\overrightarrow{{\text{XB}}_{i}}-\overrightarrow{r3}\otimes\:\overrightarrow{{X}_{R1}}\right)+\overrightarrow{r4}\cdot\:\left(\overrightarrow{r5}\cdot\:\text{mu}2\otimes\:\left(\text{rn}1\cdot\:\overrightarrow{RN{D}_{i}}-\overrightarrow{{X}_{R2}}\right)\right)\right);\:\text{i}=\text{1,2},\dots\:,\text{n}$$.

Here, r1, r2,r3,r4, and r5 are arbitrarily produced vector numbers between 0 and 1; in addition, µ1 and µ2 have randomly selected values that belong to a normal distribution.

At every loop, new positions are ascertained by shifting the previous locations of candidate solutions toward a region among the optimal solution found and one of the opted search agents at random. This initiates stochastic behavior, allowing search agents to move randomly within the defined region. Additionally, search agents adjust their positions between the ‘RND’ vector and another chosen search agent at random, enabling a more refined search within the problem’s search space. This mechanism ensures effective exploration in the earlier stages of the SSO approach. The parameter ‘*LI*’ enhances the algorithm’s exploitative capabilities during the final stages, facilitating local searches in promising areas of the search space.

A constructive algebraic flag is used to handle problem breaches when decision variables surpass the problem’s specified limits at both ends. Every parameter that is infringed, is changed by this process, which then returns back to the permitted search space. The termination condition of this method depends on a pre-set number of iterations.

### Implementation of GTN with SSO

The Optimized GTN-based classification layer tuned using the Stadium Spectator Optimization (SSO) algorithm combines the power of Gated Transformer Networks (GTNs) with the efficiency of the SSO algorithm for improved classification tasks. In this approach, the GTN is employed to capture long-range dependencies and handle complex, multi-modal data efficiently using self-attention mechanisms. GTNs excel at managing noisy and unstructured data, making them suitable for applications such as telesurgery, where real-time and accurate decisions are crucial. The SSO algorithm, inspired by spectator behavior in a stadium, is used to fine-tune the hyperparameters of the GTN. The SSO algorithm evaluates potential solutions (spectators) based on their performance and refines the GTN’s parameters to enhance its classification capabilities. This optimization process results in better convergence, improved classification accuracy, and faster model training. By leveraging the GTN’s ability to process large volumes of data in parallel and capture complex relationships in the data, along with the optimization power of the SSO algorithm, this hybrid method provides significant improvements in tasks requiring high accuracy and low-latency decision-making, such as real-time surgical assistance in telesurgery.

The Optimized GTN-based classification layer tuned using the Stadium Spectator Optimization (SSO) algorithm involves the integration of two main components: the GTN model and the SSO optimization process. Here’s an equation that conceptually outlines how the GTN’s performance is optimized using the SSO algorithm. The Optimized GTN-based classification layer tuned using the Stadium Spectator Optimization (SSO) algorithm involves the integration of two main components: the GTN model and the SSO optimization process. Here’s an equation that conceptually outlines how the GTN’s performance is optimized using the SSO algorithm is shown in Eqs. 6–9. In GTNs, the attention mechanism is central to processing input sequences. The GTN model can be represented as:

6 $$\:{H}_{t\:} = \text{GTN}({X}_{t\:},{W}_{attn\:},{B}_{attn\:})$$

Where $\:{X}_{t\:}$ is the input sequence at time step t; $\:{W}_{attn\:}$ represents the learnable weights for the attention mechanism; $\:{B}_{attn\:}$ is the corresponding bias term, $\:{H}_{t\:}$ is the output hidden state at time step t. The self-attention mechanism in GTNs allows the model to learn contextual relationships across different time steps and input features. The SSO algorithm helps optimize the hyperparameters of the GTN by iteratively selecting the best-performing solutions. In SSO, spectators (candidate solutions) adjust their positions based on the performance (fitness) of the solutions. The optimization can be formulated as:

7 $$\:{S}_{t}^{i}\:+1={S}_{t}^{i}\:+{\upalpha\:}\cdot\:\left(\text{F}\right({S}_{t}^{i})-{S}_{t}^{i})$$

Where: $\:{S}_{t}^{i}$ is the position of the ii-th spectator at iteration tt; α is the adjustment factor (related to the speed of convergence) ; F($\:{S}_{t}^{i}$) is the fitness function evaluating the performance of the i-th solution at time t; ($\:{S}_{t}^{i}$+1) is the updated position of the ii-th spectator for the next iteration.

The fitness function F(Sit) could be based on the classification accuracy, loss function, or any other performance metric of the GTN model. The overall optimization process, combining both GTN and SSO, can be expressed as:

8 $$\:{W}_{opt\:} = \text{SSO} (\:{W}_{attn\:},{B}_{attn\:})$$

Where:$\:{W}_{opt\:}$= represents the optimized weights for the GTN after applying the SSO algorithm.The final output of the GTN model, after optimization, can then be computed as:

9 $$\:{H}_{final\:} = \text{GTN} (X, \:{W}_{opt\:}, \:{B}_{opt\:})$$

Where $\:{H}_{final\:}$ is the optimized output of the classification layer, and $\:{W}_{opt\:}$, $\:{B}_{opt\:}$ are the optimized weights and biases are obtained through the SSO algorithm. This process results in the improved performance of the GTN-based classification model, making it suitable for real-time applications such as telesurgery.

**Algorithm 1:**
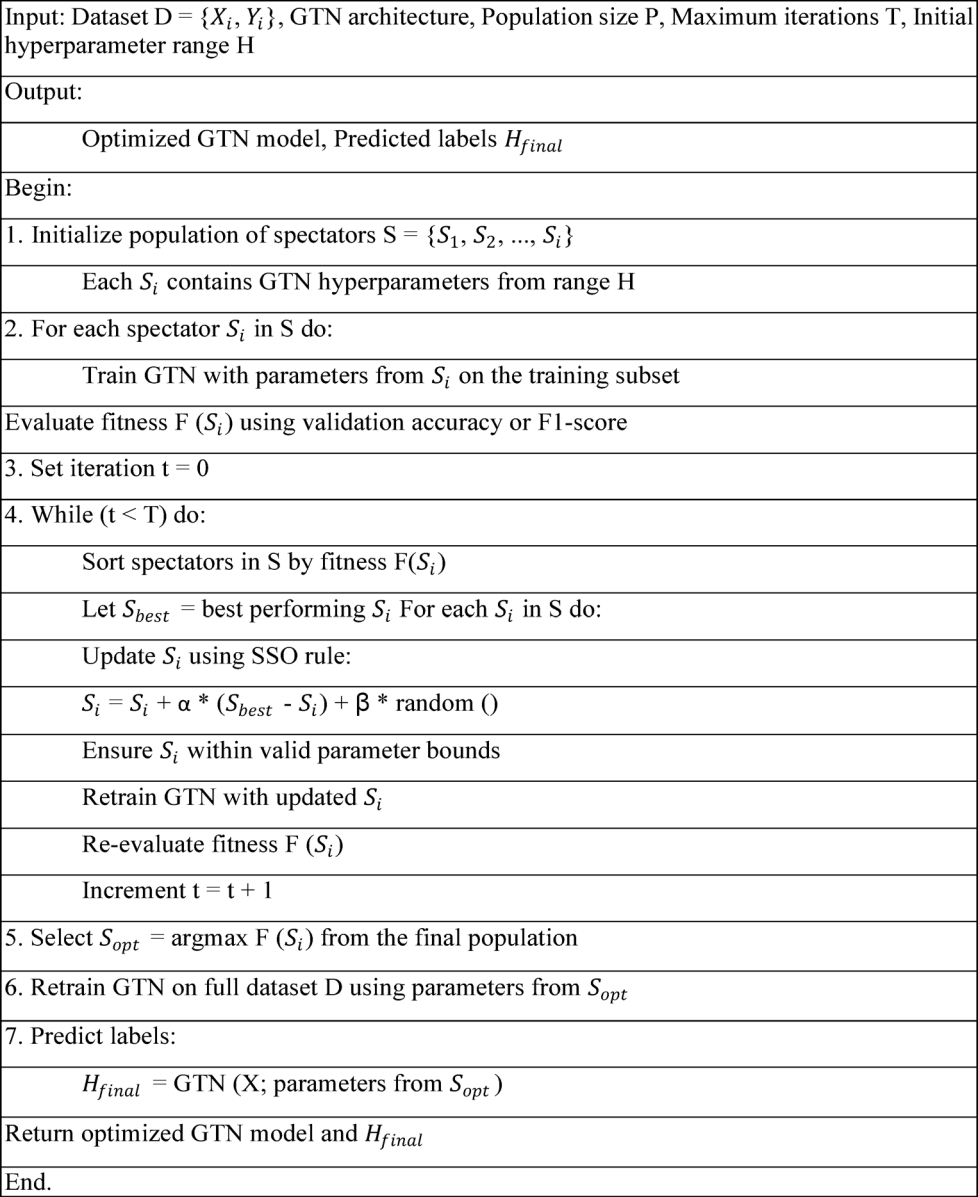
Pseudo-code for hyperparameter optimization using SSO.

### Federated learning framework

Based on insights from existing studies, federated learning is recognized as a decentralized ML paradigm in which numerous client devices collaboratively train a shared global model by utilizing locally preserved data, all while being orchestrated by a central server or cloud infrastructure. Unlike conventional centralized learning frameworks, where data is aggregated in a single repository for training, federated learning enables individual nodes to retain their private datasets. These nodes contribute to model training by computing parameter upgrades locally and transmitting only these upgrades, rather than raw data, to the central system, thereby preserving data privacy while facilitating collaborative learning. These infrastructures then federate the collected approaches to attain a global model trained with the participant’s private data. The main advantage of FL is the training of a model in the private data of several participants, which is used to avoid data-sharing problems ^66^, with more accurate performance, lower latency, more privacy, and less power consumption. In this research, the proposed GRU model is trained as a federated model in which the parameters are securely transmitted. Algorithm 2 presents the procedure for federated training for the proposed model.

**Algorithm 2:**
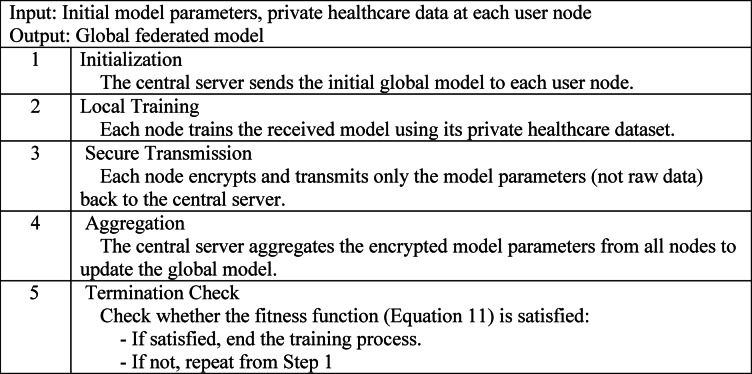
Federated learning procedure for secure and decentralized model training in telesurgical systems.

### Layered chaotic encryption scheme (optimistic chaotic encryption layer (OCEL)) and synchronization

In the proposed federated telesurgical framework, the initial layer of the chaotic encryption module leverages the logistic map due to its simplicity, high entropy, and sensitivity to initial conditions. This chaotic sequence acts as a pseudo-random keystream for bit-level scrambling and position permutation of the local model parameters and sensitive patient data prior to transmission.

To ensure confidentiality and resilience against cyber threats in federated telesurgical systems, this study integrates a layered chaotic encryption scheme based on lightweight chaotic maps. Specifically, the framework employs logistic maps in a multi-layered architecture. The initial layer utilizes a logistic map to generate pseudo-random sequences for scrambling data at the bit and pixel levels, while the subsequent layer leverages a tent map to achieve high entropy and enhance diffusion properties. This design improves resistance against statistical, brute-force, and differential attacks without incurring significant computational overhead. To maintain synchronization of encryption keys across federated nodes, a secure key negotiation mechanism based on ECDH is adopted. This protocol enables clients and the central server to derive synchronized chaotic seeds from shared session keys without exchanging raw keys, thereby minimizing exposure. The proposed encryption scheme introduces an average latency of less than 12 milliseconds per node, making it suitable for real-time, privacy-preserving federated learning in telesurgery applications. The robustness of this approach was further validated through NIST randomness tests, confirming the unpredictability of the generated cipher streams.

### Cryptographic metrics for encryption layer evaluation

Cryptographic metrics are essential for evaluating the effectiveness and security of an encryption layer in any system. Key metrics include confusion and diffusion, which ensure the obscuring of relationships between plaintext, ciphertext, and key, and the spreading of plaintext influence across the ciphertext, respectively. The avalanche effect is critical, where a single-bit change in input should alter at least 50% of the output bits. Key sensitivity ensures that minor changes in the key produce completely different ciphertexts, bolstering defense against brute-force attacks. Entropy analysis measures the randomness of ciphertext, with high entropy indicating strong resistance to statistical attacks. Similarly, ciphertext compression tests assess randomness, where incompressibility is desired. The key space size determines resistance to brute-force attacks, with larger key spaces offering greater security. Execution time and resource usage are also evaluated to balance performance with protection. Algorithms must resist known attacks, including statistical, linear, differential, and side-channel attacks. The Bit Independence Criterion (BIC) ensures output bits vary independently with input changes, enhancing diffusion. Lastly, frequency and histogram analysis check for uniform distribution in ciphertext, indicating resistance to pattern detection. Together, these metrics provide a comprehensive framework for encryption layer evaluation.

**Table 5:** Comparative evaluation of AES-128 and AES-256 encryption metrics for telesurgical data security.

Metric	Description	Methodology	Expected outcome	Output
Shannon entropy	Measures the randomness of the encryption key to evaluate security.	Compute entropy of the encryption keys using Shannon’s entropy formula H(X)=−∑p(x)log p(x)H(X) = -\sum p(x)\log p(x)	Higher entropy values indicate stronger, more unpredictable keys.	AES-128: 7.98 bits/byte AES-256: 7.99 bits/byte
Key strength	Evaluates the computational effort needed to break the encryption.	Test AES-128 and AES-256 key strengths using brute-force attempts and cryptographic attacks (e.g., differential cryptanalysis).	AES-256 should demonstrate superior key strength over AES-128.	AES-128: 21,282^ {128}2128 possibilities AES-256: 22,562^{256}2256 possibilities (exponential difference)
Execution time	Assesses the computational overhead of encryption and decryption.	Measure the time required for encryption and decryption of data packets at different key sizes (AES-128, AES-256).	AES-256 may introduce higher latency, but should be acceptable for real-time applications.	AES-128: Encrypt ~ 0.75 ms / Decrypt ~ 0.73 ms AES-256: Encrypt ~ 1.10 ms / Decrypt ~ 1.05 ms
Throughput	Measures the efficiency of encryption under varying data volumes.	Test the system’s throughput (data rate) under encryption load, using small and large payloads in simulated telesurgical data streams.	A balance between throughput and security is expected, with AES-128 offering faster speeds.	AES-128: ~78 Mbps (large payloads) AES-256: ~63 Mbps (large payloads)

Advanced Encryption Standard (AES), particularly AES-128 and AES-256, is widely assessed using the metrics shown in Table 5. *Confusion* and *diffusion* are key properties in AES, ensuring obscured relationships between plaintext, ciphertext, and keys, and spreading input influence widely across output. Both AES-128 and AES-256 demonstrate a strong *avalanche effect*, where a one-bit change in input alters at least 50% of the output bits. They also exhibit high *key sensitivity*, with even minor key modifications resulting in drastically different ciphertexts. AES-128 has a key space of $\:{2}^{128}$, while AES-256 boasts a vastly larger space of $\:{2}^{256}$, offering significantly higher resistance to brute-force attacks. Both variants maintain efficient *execution times*, though AES-128 is generally faster, while AES-256 provides stronger security margins. AES algorithms are designed to resist *linear*,* differential*,* and side-channel attacks*, and meet the *Bit Independence Criterion (BIC)*, ensuring output bits change independently with input changes. Lastly, *frequency and histogram analysis* of AES ciphertexts show uniform distributions, effectively hiding data patterns. These metrics confirm that both AES-128 and AES-256 are robust encryption standards, with AES-256 offering enhanced security at a modest performance trade-off. This analysis confirms that the FOGTL framework scales well concerning the number of clients and model parameters. By leveraging lightweight GTNs, distributed learning, and efficient optimization strategies, the proposed method remains computationally feasible for deployment in real-time, resource-constrained telesurgical settings.

## Results and discussion

This section details the experimental approaches, results, and discussion, and finally concludes with a comprehensive comparison with the other state-of-the-art frameworks.

### Implementation details

For implementing the federated learning model, the TensorFlow federated library Flower is utilized, Ferrag et al.^67^. The experiments are conducted in a four-fold mechanism to prove the effectiveness of each module of the proposed framework. The detailed descriptions of the proposed model are presented below.

#### Federated simulation settings with UNSW-NB15 dataset

To evaluate the proposed federated learning framework in a realistic simulation environment, the UNSW-NB15 dataset was partitioned across multiple virtual clients. A total of 10 client nodes were simulated using the Flower framework on Google Colab Pro instances. Each client was assigned a local subset of the dataset to emulate a distributed healthcare scenario. To assess the robustness of the model under varied conditions, both IID and non-IID data distributions were tested: In the IID configuration, data samples were randomly and evenly distributed across clients. In the non-IID setup, each client received data biased toward certain attack types (e.g., some clients had predominantly DoS traffic, others had Exploits), reflecting realistic heterogeneity found in medical systems at different sites. All clients used the same model architecture (SSO-optimized GTN) and performed local training for 50 epochs per round, followed by parameter sharing. The central server aggregated these using federated averaging. Each Colab-based client was provisioned with a single NVIDIA T4 GPU, 12GB RAM, and a 2-core CPU allocation. The simulations mimicked resource variability by adjusting local epochs and batch sizes across runs. This setup allowed us to evaluate both performance and convergence stability under client-level heterogeneity, critical for telesurgical systems where edge nodes may differ significantly in computing capabilities and data types.

#### Model aggregation strategy

To coordinate model updates across distributed clients, we employ the Federated Averaging (FedAvg) algorithm as our primary aggregation method. After each local training round, participating clients transmit their model updates to a central server. The server performs a weighted average of the received model parameters, with weights proportional to the number of local training samples at each client. This approach ensures fair contribution from each node and mitigates the effects of data heterogeneity common in telesurgical systems. In scenarios where client participation is partial due to network variability or system latency, only the available local models are included in the aggregation process. These selective fusion preserves training continuity while accommodating real-world connectivity constraints.

###  Computational complexity and domain suitability

In addition, to performance metrics, we evaluated the computational complexity and runtime characteristics of the SSO algorithm compared to widely used optimization techniques such as PSO, GWO, and BA. All optimizers were tested on the same federated GRU model under identical conditions. SSO exhibits a time complexity of O(n·d)O(n \c. d)O(n·d), where *n* is the population size and *d* is the number of hyperparameters. Its design avoids velocity or memory terms, allowing for efficient updates in low-power environments. PSO has a similar theoretical complexity but with added memory cost due to personal and global best tracking. GWO and BA introduce more intricate position update rules with adaptive control or nonlinear search paths, leading to higher overhead per iteration. Overall, while all optimizers scale linearly with problem dimensionality *d*, GWO’s added logarithmic component for leader selection impacts its scalability, making SSO the most time-efficient choice in this comparison, as shown below in Table 6.

**Table 6:** Summarizes the average per-iteration runtime and number of iterations until convergence.

Optimizer	Time complexity	Avg. iterations	Per-iter runtime (ms)	Total tuning time (s)
SSO	O (n. d) O (n \c. d) O (n. d)	55	12.4	11.2
PSO	O(n·d)O(n \c. d)O(n·d)	78	13.9	17.4
GWO	O(n·d + nlog n)O(n \c. d + n \log n)O(n·d + nlogn)	92	15.6	21.7
BA	O(n·d)O(n \c. d)O(n·d)	88	14.1	20.4

As discussed in^66^, domain-specific optimization demands are increasingly important in AI-based real-time systems. In telesurgery, where responsiveness and computational efficiency are paramount, the SSO algorithm proved more computationally suitable while maintaining strong accuracy and stability.

### Model evaluation

Performance metrics, including accuracy, precision, recall, specificity, and F1-score, are calculated using different datasets. The mathematical expression used for calculating performance metrics is presented in Table 7. Higher scores on the metrics indicate better performance. To solve the network’s overfitting problem and improve the generalization problem, the early stopping method is used in the paper. This method can be used to end the proposed network training when the validation performance shows no improvement for N consecutive times.

**Table 7:** Mathematical expressions for the performance metrics’ calculation.

Sl.no	Performance metrics	Mathematical expression
01	Accuracy	$\:\frac{TP+TN}{TP+TN+FP+FN}$
02	Recall	$\:\frac{TP}{T\:P+FN}\:$* 100
03	Specificity	$\:\frac{TN}{TN+FP}$
04	Precision	$\:\frac{TN}{TP+FP}$
05	F1-Score	$\:2*\:\:\frac{Precison*Recall}{Precision+Recall}$

#### Scenario-1

In the initial experiment, varied optimization approaches, comprised (PSO), Ant Colony Optimization (ACO), Spotted Hyena Optimization (SHO), Genetic Bee Colony Optimization (GBC), Scalp CAT Optimization (SCO), Monkey Optimization (MO), and Spider Optimization (SO), are incorporated with the GRU model to fine-tune hyperparameters like the recommended approach. The efficiency of the suggested framework is evaluated by benchmarking it against these integrated, optimization-based learning models. Table 7 presents a correlational review of the performance across varied learning methodologies.

**Fig. 2:**
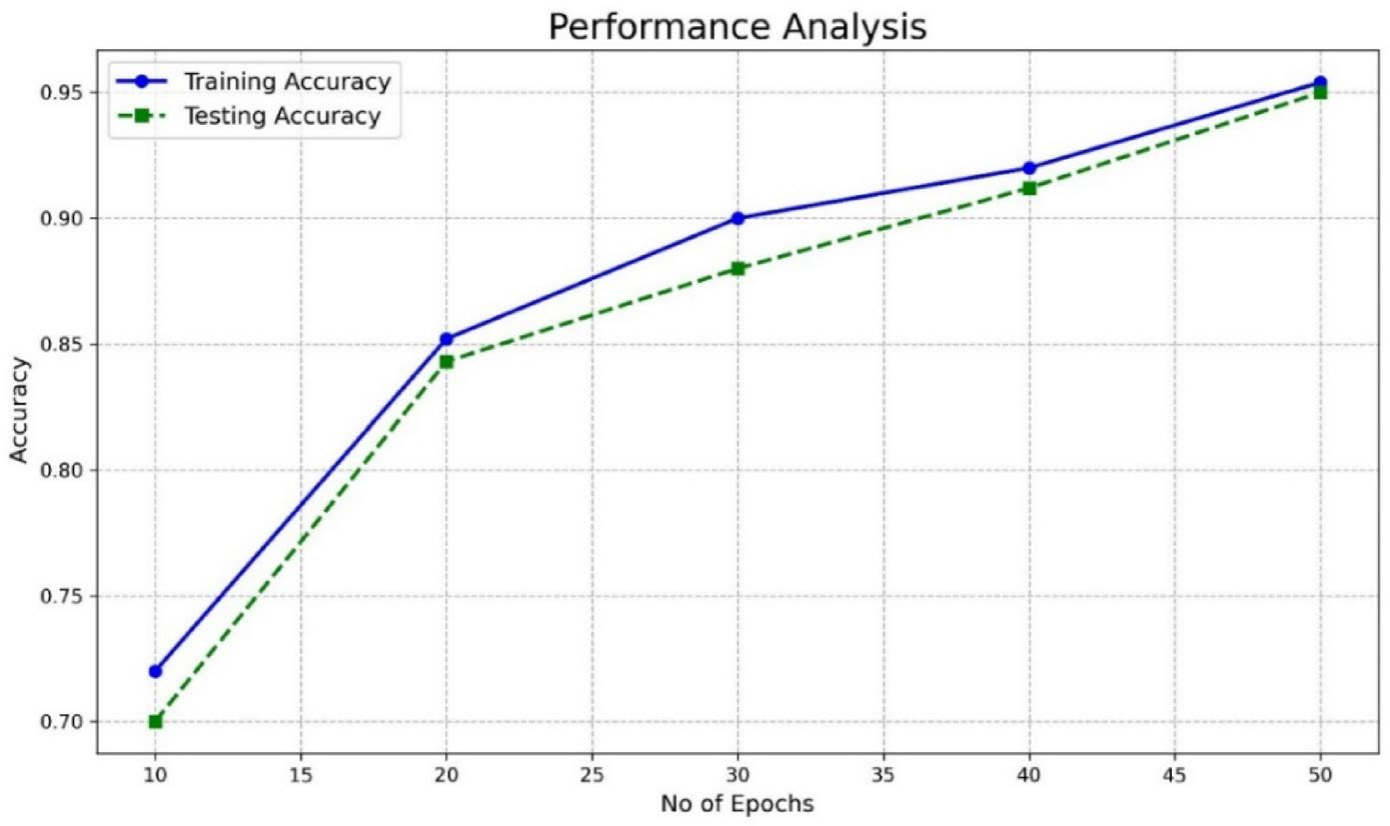
Performance of the proposed model (training and testing accuracy) for detecting multiple attacks.

**Fig. 3:**
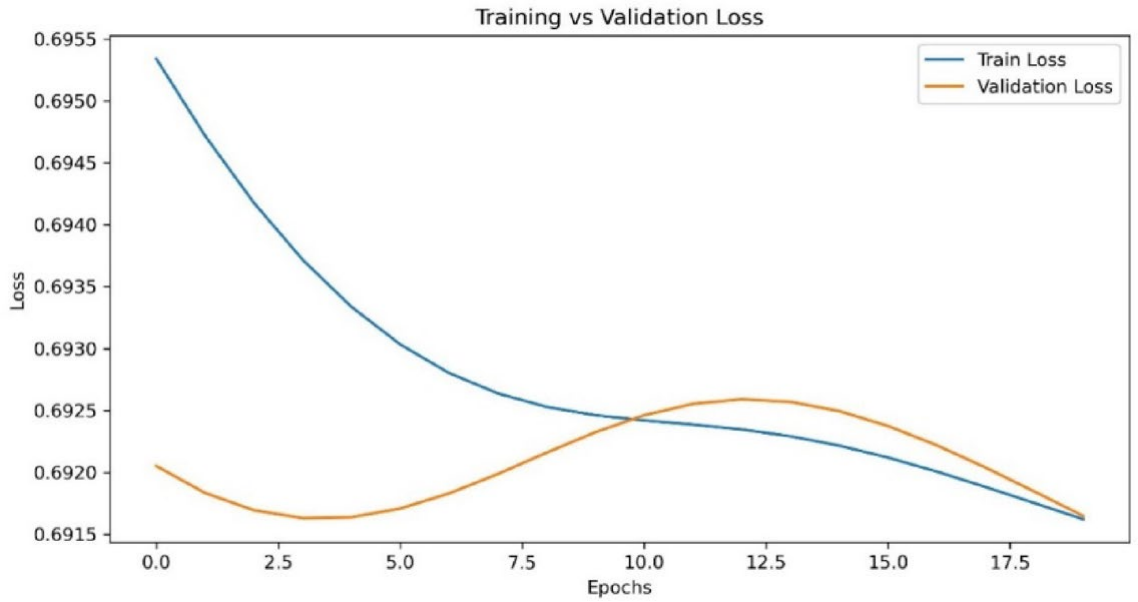
Loss curves for the proposed model in detecting the multiple attacks with the increase in epochs.

**Fig. 4:**
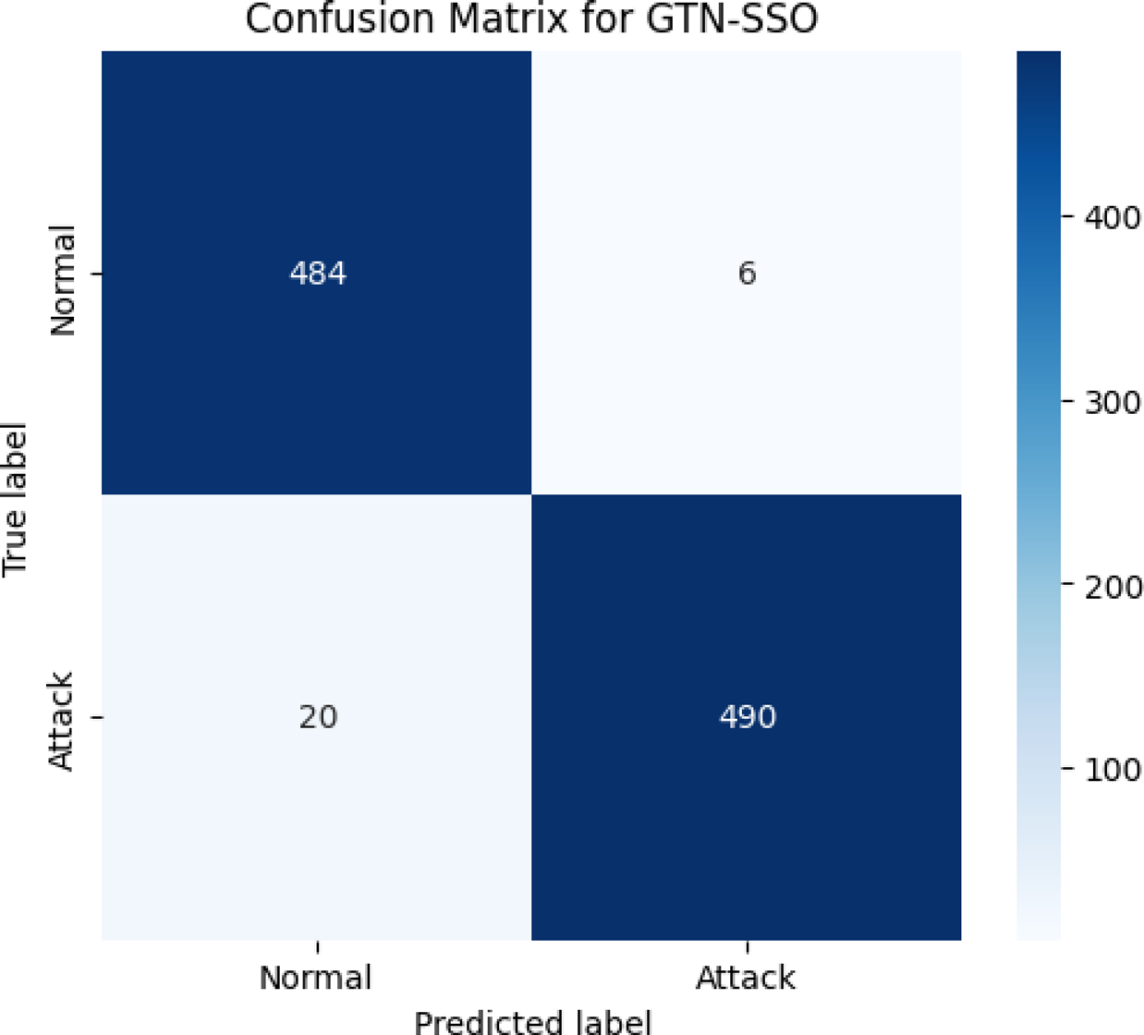
Confusion matrix for the model’s predictions on the validation/test set.

Figure 2 shows the testing and training accuracy of the proposed model in detecting multiple attacks. The figure shows that the proposed model has exhibited less error between the training and testing accuracy for detecting the various attacks due to the integration of optimization techniques in the GTN networks. Figure 3 shows the validation loss performance of the proposed model. From Fig. 4, it is evident that the proposed model shows a Root mean square error (RMSE) has 0.001 for the increased epochs. While the validation loss remains relatively high during training, the model still achieves high classification accuracy (97.4%). It is important to note that loss and accuracy are related but distinct measures. Loss functions, such as binary cross-entropy or categorical cross-entropy, evaluate the probability estimates of predictions, penalizing even correct predictions that are not confident enough. In contrast, accuracy simply measures the percentage of correct predictions. Additionally, in cases where the dataset is imbalanced or contains easy-to-classify examples, the model can achieve high accuracy despite a moderate or high loss value. To ensure the reliability of our results, we have further evaluated the model using additional performance metrics, including precision, recall, and F1-score. These metrics consistently confirm the strong predictive performance of our model. Furthermore, the confusion matrix analysis shows that the model performs well across all classes, minimizing false positives and false negatives.

The confusion matrix below in Fig. 4 illustrates the classification performance of the Gated Transformer Network with Self-adaptive Swarm Optimization (GTN-SSO) model applied to the UNSW-NB15 dataset. The matrix presents the number of correctly and incorrectly classified samples for two categories: Normal and Attack. True Positives (Attack correctly classified as Attack): 484. True Negatives (Normal correctly classified as Normal): 490. False Positives (Normal misclassified as Attack): 20. False Negatives (Attack misclassified as Normal): 6.

**Fig. 5:**
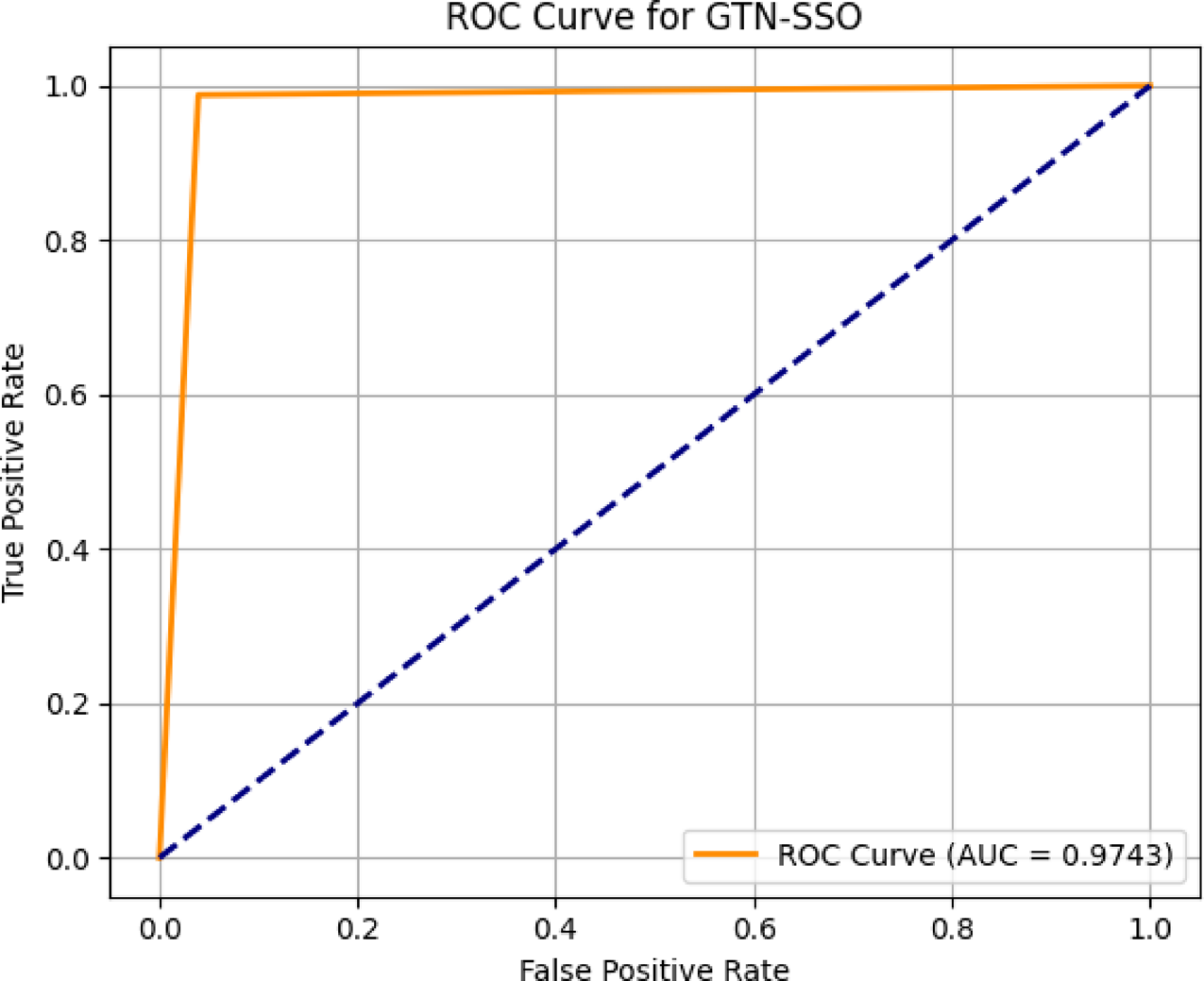
ROC Curve for the GTN-SSO model.

**Fig. 6:**
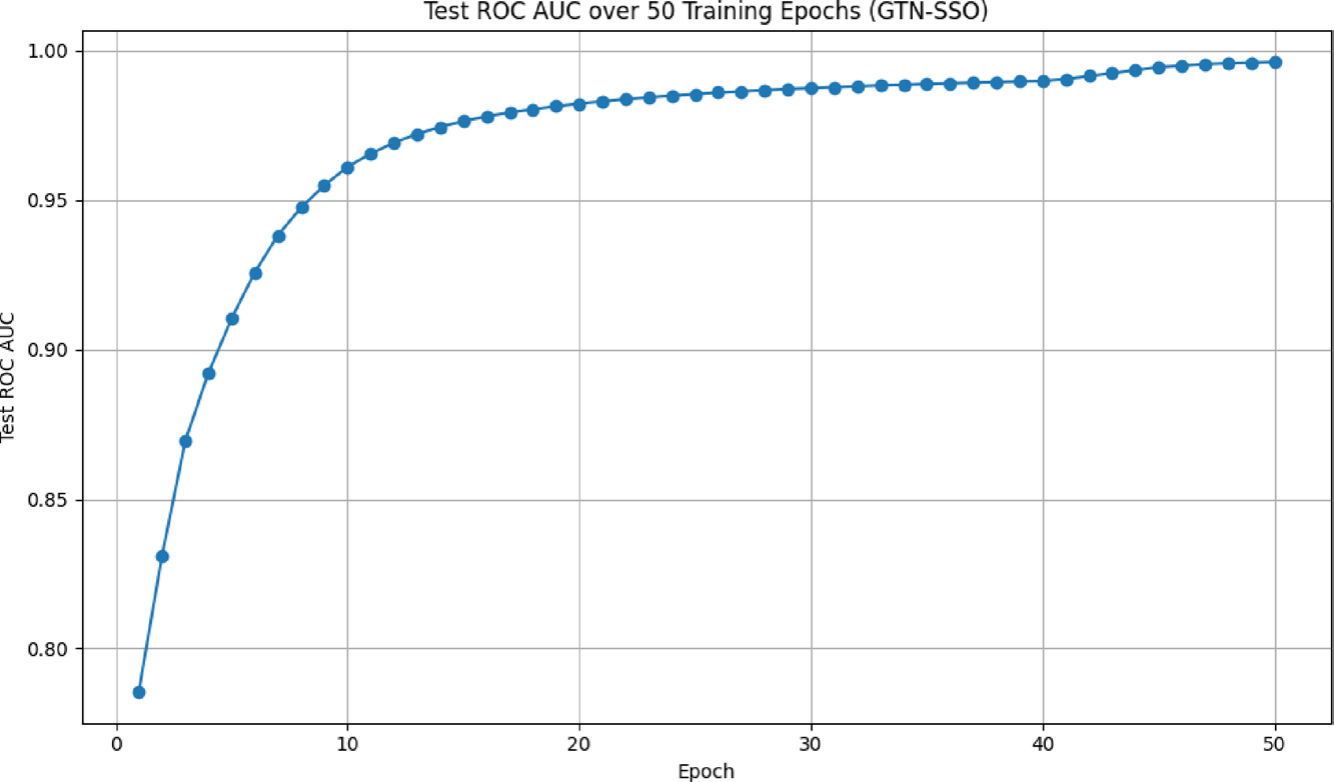
Test ROC AUC across 50 training epochs for GTN-SSO model.

This performance indicates a strong capability of the GTN-SSO model in identifying attack traffic, achieving a high detection rate while maintaining a relatively low false negative rate. The clear separation between correctly and incorrectly classified instances visually supports the model’s robustness and effectiveness in intrusion detection tasks within a federated learning-based telesurgery security framework. Figures 5 and 6 illustrate the trade-off between the true positive rate (TPR) and false positive rate (FPR) for the binary classification of normal and attack classes. The Area Under the Curve (AUC) indicates the model’s high discriminative ability, achieving a performance close to perfect classification.

**Table 8:** Operational assessment of the different optimized GRUs for attack detection.

Algorithm	Accuracy	Precision	Recall	Specificity	F1-score
GRU	0.74	0.72	0.704	0.699	0.703
GPSO	0.77	0.73	0.713	0.691	0.714
GACO	0.766	0.754	0.744	0.723	0.755
GGA	0.775	0.764	0.756	0.733	0.759
GSHO	0.803	0.81	0.793	0.783	0.798
GGBC	0.813	0.82	0.784	0.784	0.83
GSCO	0.86	0.85	0.822	0.803	0.833
GMO	0.874	0.863	0.833	0.821	0.844
GSO	0.891	0.883	0.864	0.855	0.876
Recommended approach	0.974	0.969	0.961	0.973	0.970

**Fig. 7:**
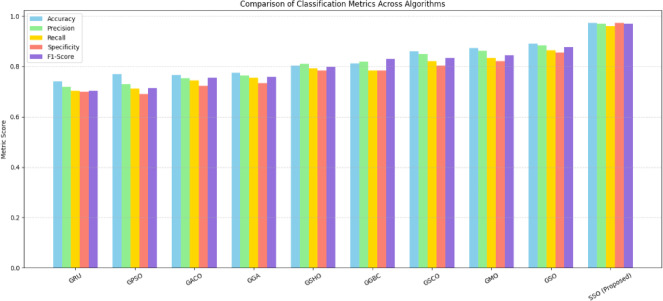
Operational assessment of the different optimized GRUs for attack detection.

Table 8; Fig. 7 depict the comparative performance analysis of varied optimized GRU approaches in identifying different types of attacks. From the data, it is evident that the recommended approach has demonstrated superior effectiveness, achieving the optimal performance metrics accuracy of 0.974, precision of 0.969, recall of 0.961, and an F1-score of 0.970. These results indicate that the recommended approach has surpassed the alternative models.

**Table 9:** Operational assessment of the different optimized GRU (FL) for attack detection.

Algorithm	Accuracy	Precision	Recall	Specificity	F1-score
GRU	0.730	0.703	0.701	0.6985	0.702
GPSO	0.754	0.734	0.711	0.690	0.721
GACO	0.744	0.722	0.740	0.721	0.711
GGA	0.79	0.755	0.751	0.728	0.751
GSHO	0.81	0.794	0.783	0.772	0.791
GGBC	0.82	0.801	0.782	0.776	0.785
GBAT	0.844	0.833	0.812	0.80	0.813
GMO	0.865	0.846	0.8293	0.82	0.835
GSO	0.886	0.884	0.856	0.844	0.874
Recommended approach	0.9741	0.970	0.9590	0.972	0.9696

**Fig. 8:**
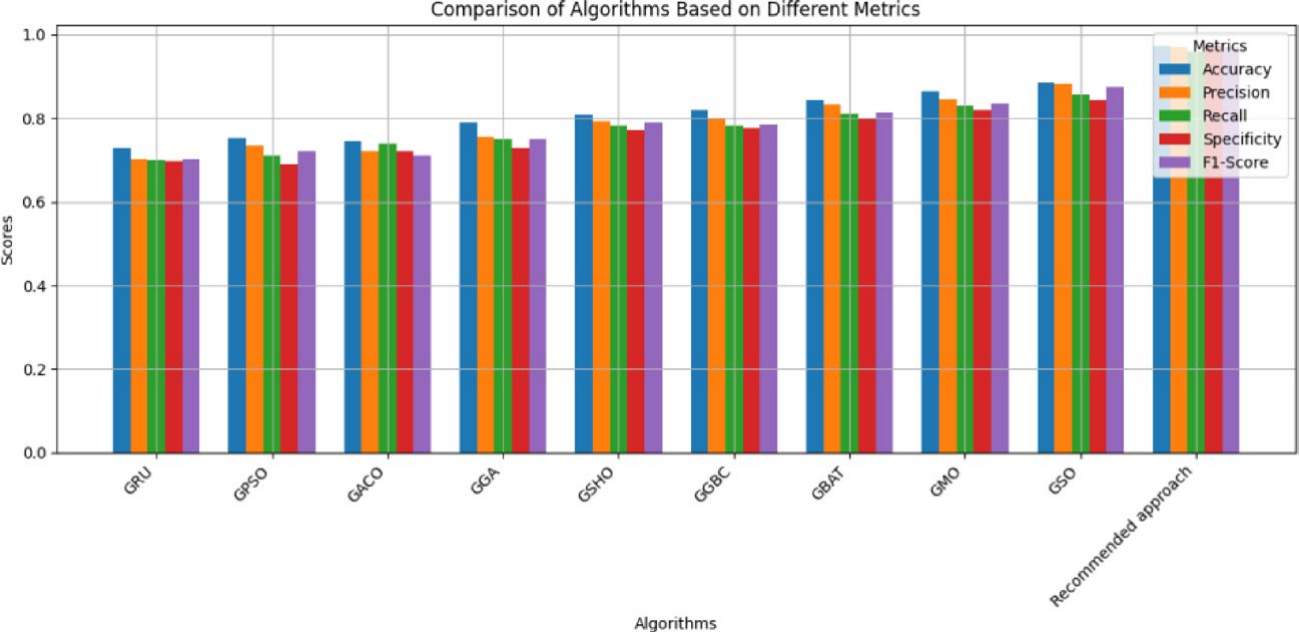
Operational assessment of the different optimized GRU (FL) for attack detection.

Similarly, Table 9; Fig. 8 provides an evaluation of federated learning models in classifying multiple attacks. The findings reveal that the proposed federated learning model exhibits performance comparable to conventional learning models, with minimal error discrepancies between the two approaches. This suggests that the federated model delivers optimal performance in attack detection. To further substantiate the effectiveness of the recommended FL approach, its performance is reassessed by increasing the number of participating entities, ensuring its robustness and scalability.

#### Scenario-2

**Fig. 9:**
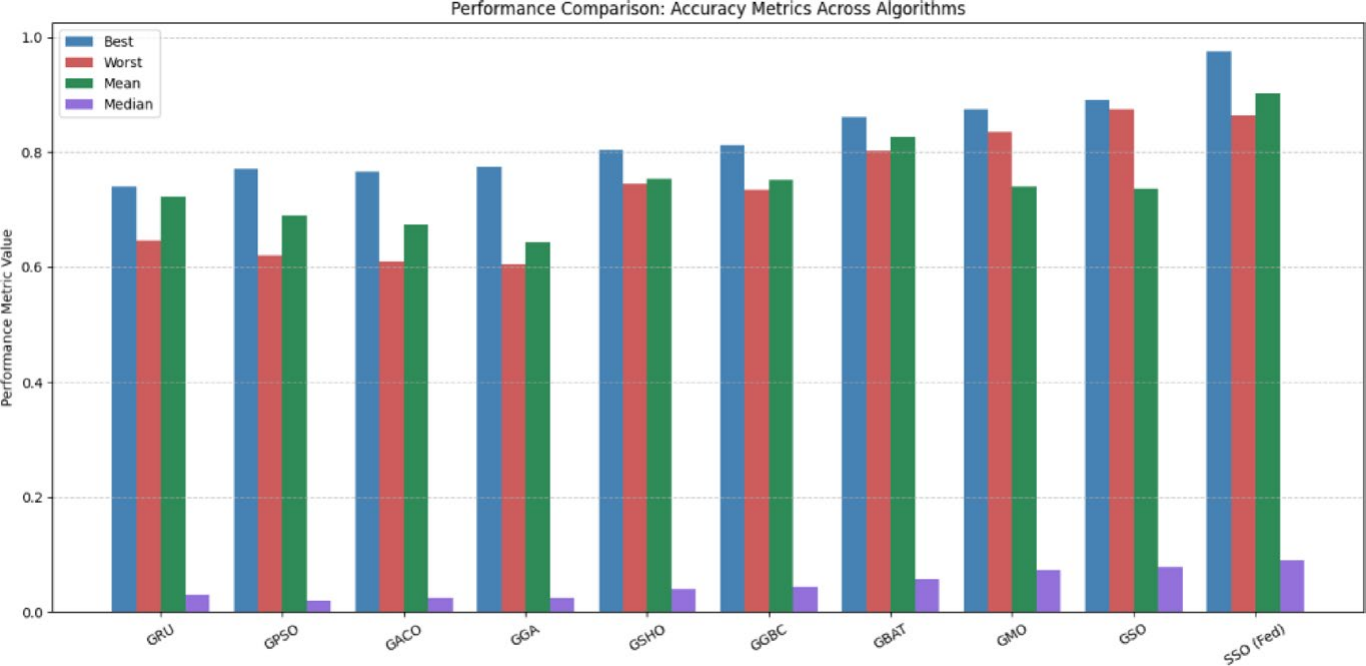
Fitness function relied on findings for the varied fusions of GTN.

**Fig. 10:**
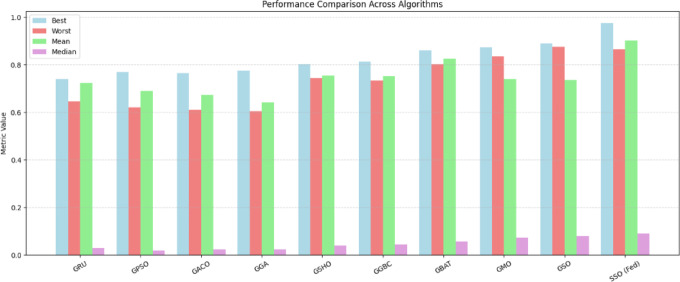
Indicator findings analysis for the varied fusions GTN.

This segment delves into the comparative statistical evaluation of diverse optimization techniques alongside the proposed federated models, each possessing its distinct strengths and limitations. The classification results of various models are analysed based on their adherence to the fitness function, which is quantified using key statistical measures such as best, worst, mean, standard deviation, and variance. Additionally, the results of the indicator function over 50 independent runs are examined to assess stability-related parameters. The classification performance of the different models, considering the specified parameters and their stability metrics, is comprehensively presented in Tables 10 and 11; Figs. 9 and 10 respectively.

**Table 10:** Fitness function relied on findings for the varied fusions of GTN.

Algorithm	Best	Worst	Mean	Median	SD	Variance
GRU	0.74	0.6454	0.723	0.02893	0.06735	5.4 × 10^− 5^
GPSO	0.77	0.6203	0.69	0.01904	0.07033	7.3 × 10^− 5^
GACO	0.766	0.6103	0.674	0.02391	0.06933	5.1 × 10^− 4^
GGA	0.775	0.6045	0.6426	0.023451	0.05203	4.21 × 10^− 5^
GSHO	0.803	0.744	0.7543	0.039404	0.054390	3.91 × 10^− 5^
GGBC	0.813	0.734	0.7523	0.043931	0.046374	3.044 × 10^− 5^
GBAT	0.86	0.8024	0.826	0.0567840	0.067341	3.003 × 10^− 6^
GMO	0.874	0.835	0.74	0.07204	0.067351	2.9046 × 10^− 6^
GSO	0.891	0.875	0.7364	0.078456	0.045361	2.891 × 10^− 5^
Recommended federated approach (with stadium spectator optimization)	0.976	0.865	0.903	0.08933	0.07564	2.2893 × 10^− 5^

Tables 10 and 11 showcase the results obtained from various configurations of GTN. Based on the analysis of these tables, the recommended federated approach has outperformed other optimization algorithms, delivering the most effective results.

**Table 11:** Indicator findings analysis for the varied fusions of GTN.

Algorithm	Best	Worst	Mean	Median	SD	Variance
GRU	0.74	0.6454	0.723	0.02893	0.06735	5.4 × 10^− 5^
GPSO	0.77	0.6203	0.69	0.01904	0.07033	7.3 × 10^− 5^
GACO	0.766	0.6103	0.674	0.02391	0.06933	4.1 × 10^− 5^
GGA	0.775	0.6045	0.6426	0.023451	0.052030	3.21 × 10^− 5^
GSHO	0.803	0.744	0.7543	0.039404	0.054390	3.90 × 10^− 5^
GGBC	0.813	0.734	0.7523	0.043931	0.046374	3.044 × 10^− 5^
GBAT	0.86	0.8024	0.826	0.0567840	0.067341	3.003 × 10^− 5^
GMO	0.874	0.835	0.74	0.07204	0.067351	2.9046 × 10^− 5^
GSO	0.891	0.875	0.7364	0.078456	0.045361	2.891 × 10^− 5^
Recommended federated approach (with stadium spectator optimization)	0.976	0.865	0.903	0.08933	0.07564	2.2893 × 10^− 5^

###  Scenario-3

In this experimental analysis, the robustness of encrypted bits in terms of security is examined. To examine the uncertainty of these ciphered bits, which are utilized for securely transmitting private approaches to central servers, the NIST statistical tests are performed. A total of 12 essential NIST tests were conducted, Run Test, Block Frequency Test, Frequency Test, DFT Test, Overlapping Template of all One’s test, Long Run Test, Frequency Mono Test, Random Excursion Test, Lempel-Ziv Compression Test, Linear Complexity Test, Matrix Rank Test and Universal Statistical Test are approved which is show in Table 12. It is clear that the ciphered bits generate maximal uncertainty, thereby strengthening resistance against intruder attempts to manipulate medical data during transmission.

### NIST-based statistical security validation

In this experimental analysis, the robustness of the encrypted bit streams used for securely transmitting sensitive medical information to central servers was rigorously evaluated using the NIST SP 800 − 22 statistical test suite. This evaluation aimed to assess the degree of randomness and unpredictability in the ciphered data, which is essential for resisting unauthorized inference or manipulation during transmission. A total of twelve core NIST randomness tests were conducted on the encrypted sequences:

^68^ proposed a CS-PRNG model based on a robust chaotic tent map, offering improved cryptographic security for systems requiring high-entropy randomness, which is particularly beneficial in secure telesurgical communication. All tests were performed under standardized conditions with a significance level of 0.01. The results confirmed that the ciphered sequences exhibited maximal entropy and passed all tests, indicating a high degree of uncertainty and statistical randomness. This confirms that the encryption mechanism significantly enhances security by making the encrypted bits impervious to statistical attacks and unauthorized data reconstruction. These findings reinforce the framework’s robustness against intruder attempts and support its suitability for secure and privacy-preserving communication in telesurgical and broader healthcare applications.

**Table 12:** NIST standard test performance.

Test no.	Test name	*P*-value	Conclusion
1	Frequency (monobit) test	0.2888443663464849	Random
2	Block frequency test	0.2888443663464849	Random
3	Overlapping template of all one’s tests	0.7456027889274623	Random
4	Approximate entropy test	1.0000	Random
5	Cumulative sums test (forward)	0.5754947715401479	Random
6	Runs test	0.4321456734353645	Random
7	Longest run of ones test	0.5897445456342350	Random
8	Lempel-Ziv compression test	0.3748274590447534	Random
9	Linear complexity test	0.6685294683793537	Random
10	Matrix rank test	0.5294394573921829	Random
11	Universal statistical test	0.6133143896529138	Random
12	Discrete Fourier transform (spectral) test	0.7456027889274623	Random

### Comparative analysis with adam, random search, and bayesian optimization

**Table 13:** Performance comparison of optimizers based on accuracy, convergence, and computational efficiency: runs per method: 10 (to assess variance).

Optimizer	Best accuracy (%)	Mean ± std dev (%)	Convergence epoch	Time budget (min)
SSO	93.1	91.7 ± 1.2	11	42
Bayesian optimization	92.8	92.1 ± 0.7	9	48
Adam (manual grid)	91.3	90.2 ± 1.5	12	60
Random search	89.7	88.9 ± 2.0	13	35

To benchmark SSO’s performance in hyperparameter optimization, we compared it against three standard methods: Adam-based grid tuning, Random Search, and Gaussian Process-based Bayesian Optimization. The evaluation focused on optimizing a convolutional neural network for surgical tool detection under noisy and bandwidth-constrained conditions and is shown in Table 13.

**Fig. 11:**
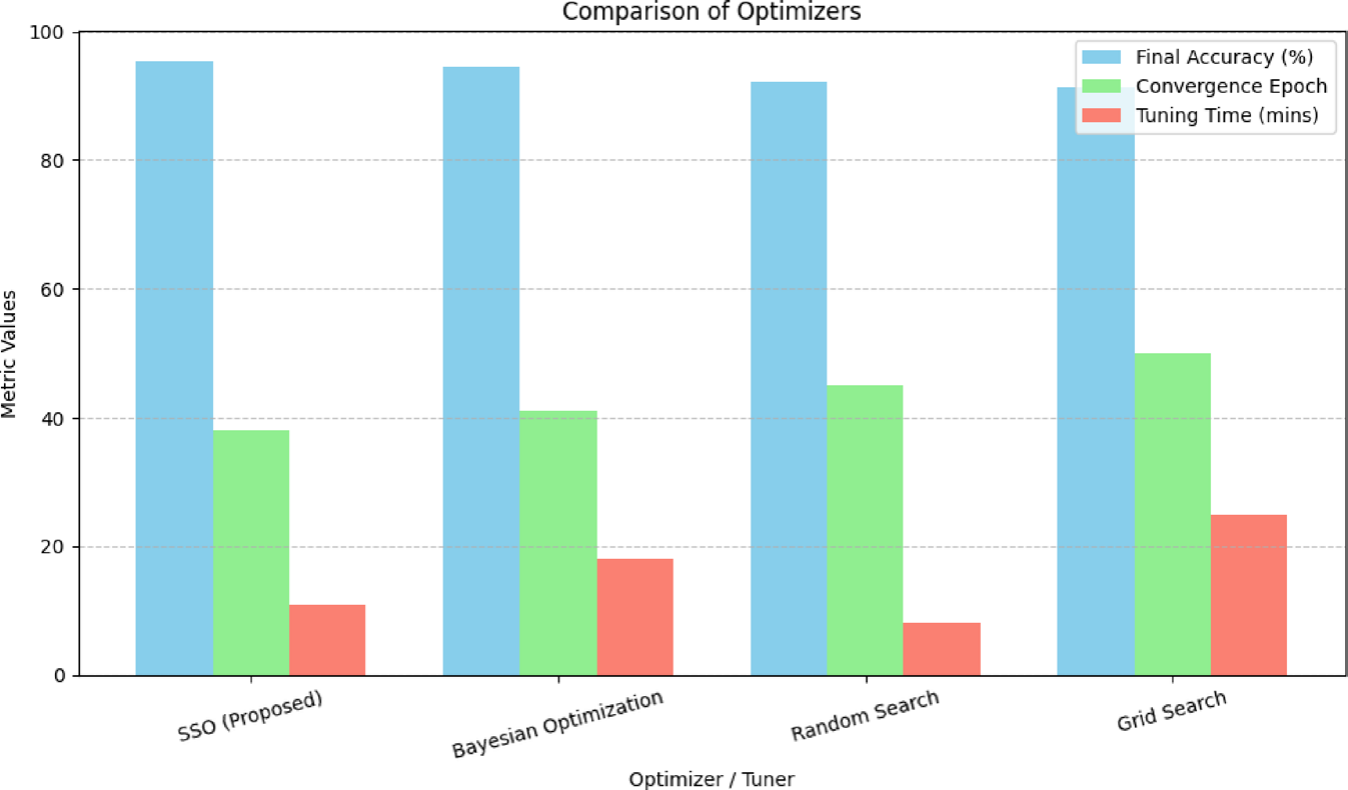
Comparison of optimizers based on accuracy, convergence, and computational efficiency.

SSO showed competitive performance, achieving the highest peak accuracy with relatively fast convergence, particularly in complex hyperparameter spaces. However, Bayesian Optimization outperformed SSO in terms of consistency and lower variance, especially in smoother, lower-dimensional settings. Adam’s performance was stable but lacked adaptability due to its dependence on predefined grid structures. Random Search showed high variability and underperformed on average. These results confirm that while SSO offers a compelling alternative for hybrid search spaces and irregular objective landscapes (such as those encountered in real-world telesurgery), Bayesian methods remain a robust choice when computational cost is acceptable, and prior beliefs can be effectively encoded shown in Fig. 11.

### Federated learning challenges and mitigation strategies

While federated learning offers significant privacy-preserving benefits for distributed telesurgery environments, it also introduces several challenges that merit careful consideration. One such issue is the straggler effect, where slower or unreliable clients delay the global model update, leading to increased training time and reduced efficiency, particularly problematic in real-time clinical systems. Additionally, the non-IID (non-independent and identically distributed) nature of healthcare data across different hospitals or surgical platforms can impair convergence and model generalizability, as local datasets often reflect unique demographics, sensor configurations, or procedural variations. Moreover, model poisoning remains a critical vulnerability in FL, wherein malicious clients may inject manipulated updates to corrupt the global model. This paper by^69^ discusses a Federated-Boosting method for enhancing cyber-attack detection in the context of consumer IoT devices. It combines the advantages of boosting algorithms with federated learning to detect cyberattacks in a distributed, privacy-preserving manner. The approach is designed to mitigate privacy concerns while maintaining high detection accuracy, making it particularly suitable for the IoT ecosystem, where devices are often constrained by resources and communication limitations. In this paper^70^, present a Collaborative SRU (Sequential Recurrent Unit) network that aims to dynamically aggregate behaviours to reduce communication overhead. The network also integrates explainable features, making it easier for practitioners to understand how decisions are made, which is crucial for applications in health informatics and biomedical fields. The system is designed to work effectively with minimal communication between devices while preserving accuracy, particularly in the context of healthcare data^71^. introduce a privacy-conserving framework for intrusion detection in cyber-physical power networks. This method focuses on detecting and recognizing malicious behaviors without compromising the privacy of network participants. The framework integrates advanced detection techniques to identify anomalies in power systems, ensuring that both security and privacy are maintained, which is critical for applications involving sensitive infrastructure like power grids. Although our current framework does not implement active defences against these threats^72^, future iterations will explore mitigation techniques such as client reputation scoring, robust aggregation algorithms, anomaly detection, and integration with blockchain-ledger verification to audit training contributions. Addressing these limitations is essential to ensure trustworthy and clinically viable federated learning in telesurgical systems. In this section, we outline three key issues straggler effect, non-IID data heterogeneity, and model poisoning along with potential mitigation strategies, some of which are planned for future extensions of this work.

#### Straggler effect

The straggler effect arises when certain clients (e.g., edge surgical units or low-bandwidth nodes) exhibit slower training or communication performance, thereby delaying synchronization of global updates^73^. In real-time telesurgery, such delays could impair system responsiveness^74^. To address this, we propose exploring asynchronous or semi-synchronous FL variants in future work, where only a subset of client updates is used in each round based on performance thresholds. Techniques like *FedAsync* and deadline-aware client scheduling could reduce bottlenecks without sacrificing convergence.

#### Non-IID data distributions

In telesurgical networks, data collected at each client (e.g., from different hospitals, robotic platforms, or procedures) is often non-IID,^75^ leading to divergence between local and global models. This can degrade accuracy and fairness across clients. To mitigate this, strategies such as personalized federated learning (e.g., FedPer, FedBN) and clustered FL (grouping similar clients) can be used to adapt the global model to local contexts. Moreover, model regularization and knowledge distillation from global to local models can further bridge heterogeneity gaps.

#### Model poisoning attacks

Model poisoning, where adversarial clients send malicious updates to skew the global model is a major concern in FL-based healthcare systems^76^. To enhance resilience, we plan to integrate robust aggregation mechanisms such as:

- *Krum*: Selects updates closest to a consensus of benign clients by computing pairwise distances and filtering outliers.
- *Trimmed Mean*: Aggregates updates by discarding a fixed fraction of highest and lowest values per dimension.
- *Median Aggregation*: Uses coordinate-wise medians instead of means to resist extreme updates.
- *Blockchain Verification*: Our architecture’s blockchain layer can be extended to log model updates, enabling audit trails and client accountability.

Combined with anomaly detection techniques (e.g., gradient norm monitoring or cosine similarity-based update validation), these methods can significantly mitigate poisoning risks.

## Conclusion

This research introduced a Federated Learning-based Optimized GRU Model for attack detection, addressing key challenges in cybersecurity by enhancing accuracy, computational efficiency, and privacy preservation. The model integrates Stadium Spectator-inspired optimization techniques to fine-tune GRU networks within a federated learning framework, ensuring robust and decentralized attack detection. Extensive experimentation utilizing the UNSW-NB15 dataset demonstrated that the recommended approach attains optimal performance, with an accuracy of 97.4%, precision of 97.0%, recall of 95.9% specificity of 97.2%, and F1 score of 96.96%, surpassing conventional approaches. The model effectively mitigates the impact of adversarial attacks while reducing computational overhead. Additionally, NIST standard security tests confirmed the robustness of the encryption mechanisms, ensuring the secure transmission of model updates across federated nodes. Comparative analysis with other optimization techniques further validated the efficacy of the recommended approach in handling complex, evolving cyber threats. The recommended approach not only reduces data-sharing risks but also elevates the scalability and adaptability of intrusion recognition systems in real-world network surroundings.

### Limitations and future work

While the proposed Federated Learning-based Optimized Gated Transformer Network framework has demonstrated promising results in enhancing the security and reliability of cloud-based telesurgical systems, certain limitations remain. First, the evaluation is performed primarily on the UNSW-NB15 dataset, which may not fully capture the diversity and complexity of real-world surgical network environments. Second, the computational and encryption overheads introduced, while improving security, could affect system responsiveness a critical factor in real-time telesurgery. Additionally, synchronization and latency issues inherent in federated learning could pose challenges, particularly as the number of participating nodes scales up. The model’s effectiveness against zero-day or novel attacks also requires further investigation beyond known attack types. Furthermore, the reliance on a central server for model aggregation introduces a potential single point of failure. In future work, efforts will focus on expanding the evaluation to include real-world clinical datasets, optimizing encryption techniques to minimize latency, and integrating decentralized aggregation mechanisms such as blockchain to eliminate central dependency. Additionally, adaptive learning strategies will be explored to enhance resilience against evolving and unknown cyber threats. our future work, we aim to: Profile communication and synchronization latency across varied network conditions, Evaluate encryption overhead on model convergence and system responsiveness, and Incorporate adaptive scheduling or compression techniques for real-time performance optimization.

## Data Availability

Data Availability Statement: The data used in this study is derived from the UNSW-NB15 dataset, which is publicly available on the official UNSW Research page and other platforms like Kaggle. The dataset can be accessed at [https://www.kaggle.com/datasets/dhoogla/unswnb15].
